# Clonal evolution and apoptosis resistance in myelodysplastic neoplasms and acute myeloid leukemia under treatment: insights from integrative longitudinal profiling

**DOI:** 10.1038/s41375-025-02756-7

**Published:** 2025-09-19

**Authors:** Paolo Mazzeo, Sarah Mae Penir, Evgenii Shumilov, Sebastian Wolf, Björn Häupl, Katharina Markus, Katayoon Shirneshan, Katharina Rittscher, Elzbieta Brzuszkiewicz, Enver Aydilek, Hannes Treiber, Thomas Oellerich, Christina Ganster, Detlef Haase, Raphael Koch

**Affiliations:** 1 Department of Hematology and Medical Oncology, INDIGHO laboratory, University Medical Center Göttingen (UMG), Göttingen, Germany; 2 Department of Meiosis, Max Planck Institute for Multidisciplinary Sciences, Göttingen, Germany; 3 Department of Hematology and Medical Oncology, University Medical Center Göttingen (UMG), Göttingen, Germany; 4 Department of Medicine A for Hematology, Oncology and Pneumology, University Hospital Münster, Münster, Germany; 5 Department of Medicine, Hematology and Oncology, University Hospital, Goethe University Frankfurt, Frankfurt am Main, Germany; 6 Frankfurt Cancer Institute (FCI), Frankfurt am Main, Germany; 7 University Cancer Center (UCT), Frankfurt am Main, Germany; 8 German Cancer Consortium (DKTK), partner site Frankfurt/Mainz, a partnership between DKFZ and UCT Frankfurt-Marburg, Germany, Frankfurt am Main, Germany; 9 German Cancer Research Center (DKFZ), Heidelberg, Germany

**Keywords:** Myelodysplastic syndrome, Translational research

## Abstract

Treatment of high-risk Myelodysplastic Neoplasms (hr-MDS) and (secondary) Acute Myeloid Leukemia (AML) remains a clinical challenge. The combination of azacitidine and venetoclax (aza/ven) may improve treatment outcomes, but still fails in a significant fraction of patients. We established a single-center collection of longitudinal samples from patients with MDS and AML/sAML and performed comprehensive genetic, proteomic and functional apoptosis profiling to identify biomarkers and targetable escape mechanisms to aza/ven. Baseline genetic characterization (*n* = 55) identified high-risk genetic alterations, while longitudinal analyses (*n* = 268, mean 8.7 [3–20] timepoints) revealed distinct genetic profiles of clonal evolution. Functional BH3-profiling at treatment initiation identified heterogeneous dependencies on BCL-2 family members. Notably, high BCL-2 dependence correlated with genetic response to aza/ven and improved overall survival, whereas increased BCL-xL dependence was associated with resistance. We further identified patterns of acquired resistance, with loss of apoptotic priming and shifts in anti-apoptotic dependencies contributing to treatment failure. BH3 profiling revealed functional shifts toward MCL-1 and/or BCL-xL in individual cases, suggesting potential therapeutic targets to overcome resistance. In vitro, BCL-xL inhibition effectively counteracted resistance in increased BCL-xL dependence cases. In summary, we characterized treatment-associated clonal evolution in MDS and AML, providing insights into clinical response, disease progression and potential individualized therapeutic strategies.

## Introduction

Myelodysplastic neoplasms (MDS) and Acute Myeloid Leukemia (AML) are malignancies arising from transformed hematopoietic stem cells by multiple genomic events, deregulated apoptosis and immune dysfunction [1–4]. In MDS, these factors lead to ineffective hematopoiesis, dysplasia and an increased risk of progression to AML with poor prognosis [5, 6].

While low‑risk MDS is managed primarily with supportive care, high‑risk MDS (hr‑MDS) requires therapies that modify disease course and prevent secondary AML (sAML). Allogeneic stem cell transplantation is considered the only potentially curative treatment option. However, many patients do not undergo transplantation due to advanced age, comorbidities, or treatment-related risks [7]. For these patients, palliative treatment with hypomethylating agents (HMA) such as azacitidine (aza) and decitabine is an available therapy to improve symptoms and prolong survival [8–11]. The therapeutic efficacy of HMAs, though, is challenged by primary refractoriness but also by high relapse rates primarily driven by clonal evolution. These limitations underscore the urgent need for innovative therapeutic strategies.

Comprehensive genetic profiling of MDS and AML recently led to the development of molecularly-informed risk stratification and identification of targetable genetic alterations [12, 13]. Additional studies have clarified the role of clonal evolution as a key driver of disease progression in MDS and sAML [14–17].

Besides genetic alterations, evasion of mitochondrial apoptosis is a hallmark of clonal cells in AML and anti-apoptotic BCL-2 family members have gained interest as crucial pro-survival factors and potential drug targets [18–20]. Indeed, targeting the anti-apoptotic BCL-2 protein with the BH3 mimetic venetoclax (ven) in combination with aza significantly improved treatment outcomes for older AML patients [21]. Furthermore, both pre-clinical and early clinical data show promising results for azacitidine in combination with ven (aza/ven) in hr-MDS [22, 23].

Mechanistically, aza/ven seems to leverage synergistic effects to inhibit both anti-apoptotic pathways and mitochondrial oxidative phosphorylation in AMLs, leading to a metabolic crisis in AML blasts [24, 25].

Still, the genetic background of dysregulated apoptosis throughout clonal evolution of MDS, its impact on the therapeutic efficacy of aza/ven and reliable biomarkers remain largely elusive, preventing rational patient selection and optimized therapeutic strategies [26].

We here address the issue of treatment-related clonal evolution in MDS and AML. We established a unique monocenter sample collection of 55 patients with MDS and AML and applied an integrated multi-platform approach, including comprehensive genetic and functional characterization. This approach aimed to identify predictive markers and therapeutic vulnerabilities in MDS and AML treated with aza/ven and provide a basis for optimized therapeutic strategies.

## Material and methods

A comprehensive description of the patient cohort, cell lines, genetic analyses, BH3 profiling, in vitro cytotoxicity assays, proteomic workflow, and statistical methods is provided in the Supplementary Methods. Additionally, Supplementary Tables 1 and 2 summarize the FISH probes included in the FISH panel and the genes included in the 53-gene targeted NGS panel, respectively.

## Results

### Clinical and genetic characteristics of the patient cohort

The main demographic, hematological, and clinical data from the patients are summarized in Table 1 and Supplementary Figs. 1 and 2.

**Table 1:** Baseline demographic, hematological and clinical data from the MDS/AML cohort.

Variable	All Patients, *n* = 55	
**Demographics**		
Gender (M/F), *n* (ratio)	29/26	1.12
Median age, years (range)	72	33-87
**Cytomorphologic subtypes (*n ***= **55)**		
MDS	23/55	42%
MDS-IB1/2	14/23	61%
MDS-LB	8/23	35%
MDS-*SF3B1*	1/23	4%
AML	32/55	58%
AML with myelodisplasia-related gene mutations	2/32	6%
AML with myelodisplasia-related cytogenetic abnormalities	4/32	13%
AML with mutated *NPM1*	3/32	9%
AML with mutated *TP53*	1/32	3%
AML NOS	2/32	6%
Progression to AML		
AML with myelodisplasia-related gene mutations, progressed from MDS/MPN	11/32	34%
AML with myelodisplasia-related cytogenetic abnormalities, progressed from MDS/MPN	4/32	13%
AML with mutated *TP53*, progressed from MDS	1/32	3%
AML NOS, progressed from MDS	1/32	3%
Progression to AML, therapy-related		
AML with *MLLT3::KMT2*, therapy-related	2/32	6%
AML with mutated *TP53*, therapy-related	1/32	3%
**Prognostic risk categories**		
IPSS-M (*n* = 23)		
Low	3/23	13%
Moderate low	2/23	4%
Moderate high	2/23	4%
High	7/23	30%
Very high	9/23	39%
IPSS-R (*n* = 23)		
Low	5/23	22%
Intermediate	4/23	17%
High	6/23	26%
Very High	8/23	35%
IPSS-R Cytogenetic risk groups (*n* = 21)		
Good	14/21	67%
Intermediate	1/21	5%
Poor	2/21	9%
Very poor	4/21	19%
ELN risk groups (*n* = 32)		
Favorable	1/32	3%
Intermediate	15/32	47%
Adverse	16/32	50%
**Blood counts, median (range)**		
Hemoglobin (g/dl)	9.4	4.9-12.6
Platelets (10^9^/L)	50.0	6.0-525.0
ANC (10^9^/L)	0.85	0.02-19.4
Bone marrow blasts (%)	15.0	0-77.0
Peripheral blood blasts (%)	7.5	0-80.0
**Cytogenetics (*n*** = **49)**		
Normal karyotype, *n* (%)	26	53%
Altered karyotype, *n* (%)	23	47%
Complex karyotype, *n* (%)	10	20%
**Treatments from enrollment (*n*** = **55)**		
Aza monotherapy (*n*, %) number therapy cycles, median (range)	20 2,5	36% (1-23)
Decitabine (*n*, %) number therapy cycles, median (range)	2 1,5	4% (1-2)
aza/ven (*n*, %) number therapy cycles, median (range)	35 3	64% (1–17)
Decitabine+Ven (*n*, %) number therapy cycles, median (range)	1 3	2%-
Gilteritinib+Ven (*n*, %) number therapy cycles, median (range)	2 1,5	4% (1-2)
DLI (*n*, %) number therapy cycles, median (range)	9 2,5	16% (2-6)
Allo-SCT	29	53%
Allo-SCT before therapy	20/55	36%
Allo-SCT after therapy	13/55	24%
Number Allo-SCT before therapy, median (range)	0	0-2
MDS patients underwent Allo-SCT	11/29	38%
AML patients underwent Allo-SCT	18/29	62%
**Outcome (*n*** = **55)**		
Median follow-up, median mo (range)	11	1-56
Death	37	66%
OS from administration of Aza±Ven, median mo (95% CI)		
MDS (*n* = 23)	14	11-nr
AML (*n* = 32)	9	5-nr

Shown are median and range for continuous variables and absolute number and percentage for categorical variable.

*M* male, *F* female, *MDS* myelodysplastic neoplasms, *MDS-IB* myelodysplastic neoplasms with increased blasts, *MDS-LB* myelodysplastic neoplasms with low blasts, *AML* acute myeloid leukemia, *g* gram; *dl* deciliter, *µL* microliter, *ANC* absolute neutrophil count, *Aza* azacitidine, Ven Venetoclax, *DLI* donor lymphocyte infusion, *Allo-SCT* allogeneic stem cell transplantation, *aza/ven* azacitidine in combination with venetoclax, *Aza±Ven* azacitidine monotherapy or in combination with venetoclax, *nr* not reached.

**Fig. 1:**
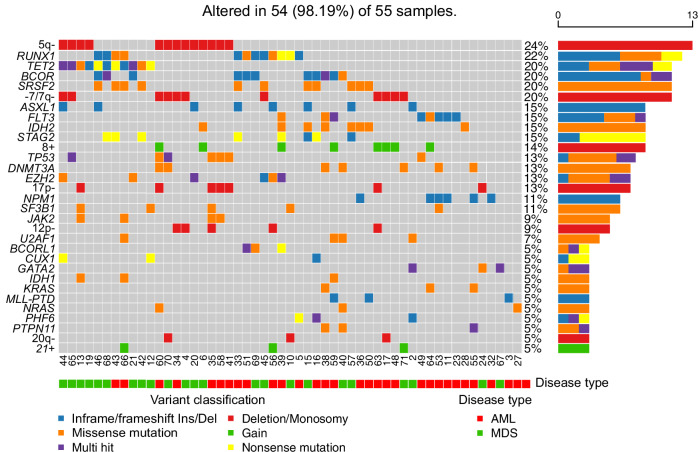
The oncoprint diagrams of mutational profile at baseline of the 55 patients. Bottom panel: Bottom panel: Disease type (AML, MDS) and variant classification are shown for each patient. Right panel: The number of times each aberration has been detected is shown as a horizontal bar, with distinct colors for each variant type. Corresponding percentage are indicated numerically to the right. Middle panel: Each column represents one patient, and each row corresponds to a mutated gene or chromosomal aberration. Color coding indicates the type of genetic alteration. Internal patient code is displayed below the matrix. AML acute myeloid leukemia, MDS myelodysplastic neoplasms.

Cytogenetics, including chromosome banding analysis (CBA) and/or fluorescence in situ hybridization (FISH), and molecular genetics were available for all patients. Karyotype based on banding analysis at recruitment was available in 49/55 patients: normal karyotype was detected in 26/49 patients (53%) and complex karyotype in 10/49 patients (20%). Sequencing was performed in all patients and somatic mutations were identified in the analyzed genes in 48/55 (87%) patients. The most common aberration was del(5q) in 13/55 patients (24%); *RUNX1* mutation in 12/55 (22%); *TET2-, BCOR*-, *SRSF2*-mutation and monosomy 7/del(7q) in 11/55 patients each (20%); *ASXL1*-, *FLT3* (ITD/TKD), *IDH2-*, *STAG2-*mutation and trisomy 8 in 8/55 each (15%); *DNMT3A-*, *TP53-* and *EZH2*-mutation and del(17p) in 7/55 each (13%). All genetic aberrations at diagnosis are shown in Fig. 1. Furthermore, we performed copy number variation analyses (CNVs) on a subset of 13 pts characterized by whole-exome sequencing (WES), which revealed amplifications and deletions in additional chromosomal regions beyond the applied CBA and FISH panel (Supplementary Fig. 3).

Together, the genetic alterations identified in our cohort represent the typical genetic landscape of high-risk myelodysplastic neoplasms (hr-MDS) and acute myeloid leukemia (AML).

Addressing factors associated with survival, we performed multivariate analyses (Supplementary Table 3). Here, BM blasts percentage at recruitment [1.04 (1.01–1.07), *p* = 0.024] and mean Bcl-2 dependencies prior to initiation of treatment for the patients who received aza/ven therapy [0.03 (0.00–0.44), *p* = 0.012] were significantly associated with overall survival (OS). Notably, BM blasts emerged as an independent prognostic factor in the multivariate model after adjusting for potential confounders such as BCL-2 dependence, treatment regimen and genetic risk factors. Nevertheless, the clinical relevance of this association warrants cautious interpretation, as statistical significance does not necessarily imply a strong prognostic impact.

### Proteomic signatures define functional alterations in MDS/AML

**Fig. 2:**
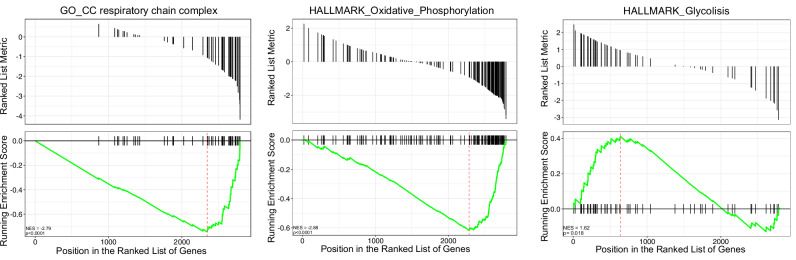
GSEA plot. Gene set enrichment analyses of differentially expressed proteins in MDS/AML patients vs. healthy donor controls.

Based on the characteristic, but heterogeneous genetic profiles of our patient cohort, we next performed global quantitative mass spectrometry (MS)-based proteomics to characterize the proteomic features of MDS/AML and compare them to CD34+ hematopoietic progenitor cells from healthy donors. We performed proteome quantification of MDS and AML samples using a sensitive high-throughput single-run workflow [27]. In this study, we identified 3500 distinct proteins in MDS/AML cells at a peptide and protein false discovery rate (FDR) of less than 5%. Supplementary Fig. 4 shows proteins differentially expressed (DEPs) in MDS/AML cohort compared to healthy donor control samples. To gain granular insight into the biological processes underlying differential protein expression, we used Gene Set Enrichment Analysis (GSEA) and the gene ontology (GO) database. This method allowed us to assess whether predefined sets of genes, associated with specific biological pathways, were enriched within the ranked list of DEPs. Notably, we observed downregulation of oxidative phosphorylation (OXPHOS) and respiratory chain complex together with upregulation of glycolysis-related pathways when comparing our cohort of MDS/AML patients to healthy control samples (Fig. 2).

### Genetic signatures of clonal evolution

To characterize genetic alterations during clonal evolution, we performed comprehensive longitudinal cytogenetics and NGS diagnostics in 30 patients. As described in Supplementary Table 4, we identified multiple high-risk genetic alterations occurring during clonal evolution, including mutations in *NRAS*, *KRAS*, *TP53*, *BCOR*, *RUNX1* and *FLT3* as well as cytogenetic abnormalities such as 16q– and 17p-, consistent with adverse-risk features per 2022 ELN and IPSS-M criteria.

**Fig. 3:**
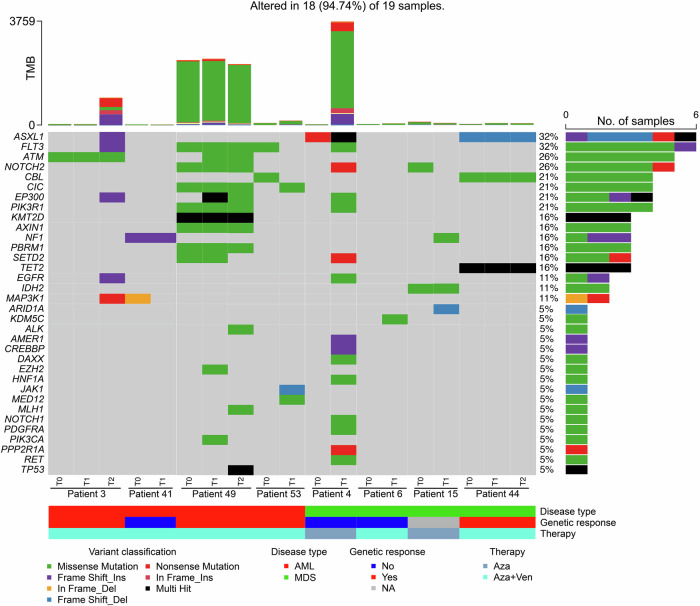
Oncoprint diagrams for WES data. Oncoprint of whole-exome sequencing performed on longitudinal samples from eight patients, annotated with disease type, genetic response as defined by decrease in clone size or variant allele frequency and applied treatment. Right panel: the percentage of patients affected by each aberration. Middle panel: the matrix of aberrations in a selection of frequently mutated genes and chromosomal aberrations. Each column represents one patient, and each row represents one aberration. AML acute myeloid leukemia, MDS myelodysplastic neoplasms, Aza azacitidine, Ven venetoclax, TMB tumor mutational burden. TMB defines the total number of non-synonymous somatic mutations per sample based on whole-exome sequencing.

Among 19 patients with either *IDH1/2* (*n* = 11) or *FLT3* (*n* = 8) mutation, 14 (74%) received aza/ven and five (26%) received aza alone. Response data were available for 10 patients in the aza/ven-treated group, of whom nine responded (9/10 patients, 90%). In contrast, among the three evaluable patients in the aza-only group, only one (1/3 patients, 33%) achieved a response. For patients with available material and clear clinical progress under treatment, but without any evidence of clonal evolution (using our custom-targeted NGS panel and FISH panel), we performed whole-exome sequencing (WES) to identify potential novel mutations or genetic alterations that might explain resistance mechanisms or disease progression. In total, we screened 13 patients by WES, collecting 25 samples from both peripheral blood (PB) and bone marrow (BM) after CD34 immunomagnetic enrichment. Longitudinal samples were obtained from 8 of these patients, resulting in a total of 19 samples. Among them, 10/19 were obtained from the enriched CD34+ cell population. The analysis identified additional genetic alterations associated with clonal evolution in six patients. We identified newly emerging mutations in genes that were not covered by our targeted NGS panel such as *ATM, NOTCH2, EP300, ARID1A* and *MAP3K1* and also noticed an increase in tumor mutational burden (TMB) in some cases during progression (Fig. 3). Remarkably, no *BCL2* G101V mutations [28] were detected in the cohort by WES analysis.

### BH3 profiling identifies biomarkers of response and resistance to aza/ven

**Fig. 4:**
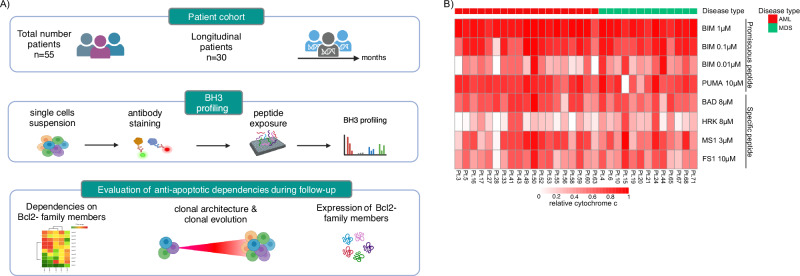
Study workflow and BH3 profiling prior treatment initiation. **A** Study workflow for patient selection and BH3 profiling. **B** Results of BH3 profiling as relative cytochrome c release in response to the BH3 peptides. BIM assesses the functionality of BAX/BAK; PUMA is a pan-sensitizer (as well as contributing to activating BAX and BAK) BAD reflects BCL-2/BCL-xL/BCL-W; HRK indicates BCL-xL dependence; MS1 targets MCL-1; FS1 targets BFL1. AML acute myeloid leukemia, MDS myelodysplastic neoplasms.

To dissect potential biological determinants of targetable anti-apoptotic mechanisms in our MDS/AML cohort, we performed flow-cytometry-based BH3 profiling (Fig. 4A). Synthetic pro-apoptotic BH3-peptides were applied to permeabilize MDS/AML blast cells (defined by CD45 lo-mid/SSC-low and CD34 expression) to assess the overall apoptotic priming and to specifically evaluate the impact of the main anti-apoptotic BCL-2 family proteins on cell survival. The flow cytometry gating strategy is depicted in the Supplementary Fig. 5.

The specific immunophenotypic profiles for the patients included in the BH3 profiling analysis are summarized in Supplementary Table 5.

BIM and PUMA were used for the overall level of priming; BAD for BCL-2/BCL-xL and BCL-W dependence; HRK for BCL-xL; MS1 for MCL-1 and FS1 for BFL1. Subtracting cytochrome c release caused by HRK from that caused by BAD (BAD-HRK) provides an index of selective BCL-2 dependence [29, 30]. DMSO and the peptide antibiotic alamethicin were used as negative and positive controls, respectively. Figure 4B illustrates the relative mitochondrial cytochrome c release in response to BH3-peptides across samples performed in 32/55 (58%) patients at a pretreatment timepoint. In summary, low concentrations of BIM (0.01 µM) revealed heterogeneity in apoptotic priming i.e. the cell’s capability to undergo apoptosis. Furthermore, peptides targeting specific anti-apoptotic BCL-2 family members (BAD, HRK, MS1, FS1) revealed highly heterogeneous dependencies on BCL2, BCL-xL, MCL1 and BFL1 (Fig. 4B). Notably, no differences across diseases (MDS and AML) were observed for all the BH3 peptides (Supplementary Fig. 6).

These findings led us to further explore the relevance of BH3 profiling in our cohort of patients treated with aza/ven (*n* = 22). All details about the cohort of 22 patients are summarized in Supplementary Table 6.

**Fig. 5:**
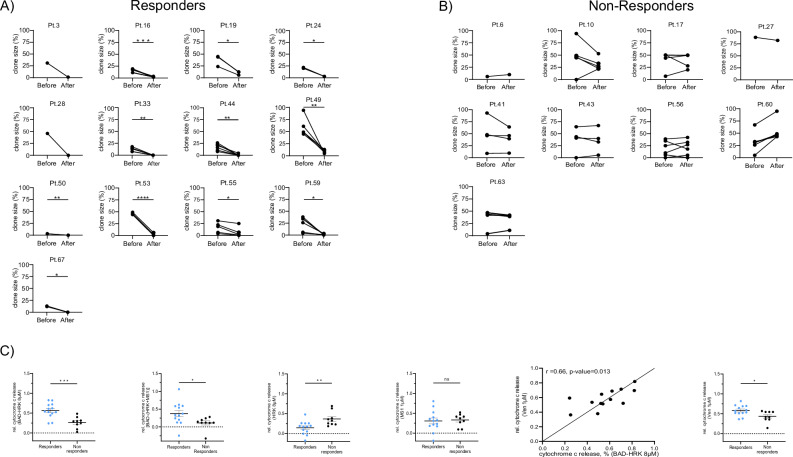
Functional apoptotis profiling in genetic responders versus non-responders. Changes in clone sizes and variant allele frequencies in patients responding to aza/ven (**A**) versus patients not responding to aza/ven (**B**). **C** BH3 profiles with relative cytochrome c release upon exposure to specific peptides in patients showing a genetic response versus non-responders. BCL-2 dependence was derived by subtracting the effect of HRK (specific for BCL-xL) from that of BAD (interacting with both BCL-2 and BCL-xL). To better isolate BCL-2-specific priming, the effect of HRK + MS1 was subtracted from the effect of BAD.

Given the established mitochondrial basis for the clinical synergy of aza/ven in literature [26], we investigated whether BH3 profiling could predict genetic response to the clinically relevant aza/ven combination. On a genetic level, we identified patients responding to aza/ven by a ≥ 50% reduction of clone sizes by FISH and/or a decrease of ≥10% VAF of driver mutations versus pts not showing such a response (Fig. 5A, B). Comparing BH3 profiles of responders versus non-responders identified significant differences in functional dependencies of specific anti-apoptotic BCL-2 family members. Assessing functional BCL-2 dependence, the relative cytochrome c release to BAD-HRK was 56.2% for the responders vs 26.4% for the non-responders group (*p* < 0.001; Fig. 5C). To further refine BCL-2-specific priming estimates, we additionally calculated BAD-(HRK + MS1), subtracting both BCL-xL and MCL-1 contributions. This composite measure showed a significant difference between responders and non-responders to aza/ven (*p* = 0.036), reinforcing the predictive value of BCL-2 dependence.

Incubation with the compound BCL-2 antagonist ven, used the same way as for BH3 peptides, produced similar results with decreased cyt c release in the responders group (*p* = 0.008; Fig. 5C). Mitochondrial cyt c release in response to ven correlated with release caused by BAD-HRK (*p* = 0.013; Fig. 5C) in the responders group, indicating on-target activity and performed well as a binary predictor of response to ven by Pearson correlation analysis (*r* = 0.66, *p* = 0.013). In contrast, high functional dependence on BCL-xL (assessed by HRK) at diagnosis was significantly associated with non-responders to aza/ven (*p* = 0.017). Notably, functional dependence on MCL1 (assessed by MS1, 37.3% vs 30.7%; *p* = 0.536) and BFL1 (assessed by FS1, 53.4% vs 48.5%; *p* = 0.651; data not shown) at diagnosis was not significantly different in responders versus non-responders (Fig. 5C).

**Fig. 6:**
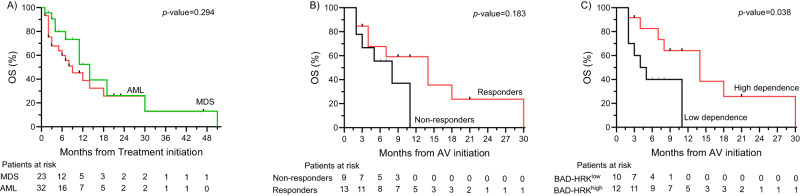
Overall survival. Overall survival of **A** the entire study cohort, discriminated by disease type, **B** pts treated with aza/ven, discriminated by genetic response versus no response, **C** pts treated with aza/ven, discriminated by high versus low BCL-2 dependence assessed by BH3 profiling using the BAD-HRK difference.

Based on these differences in functional dependencies on anti-apoptotic proteins at the initiation of treatment with aza/ven, we next explored differences of survival in these patients. Overall survival in our cohort was not significantly different in patients with MDS versus AML (*n* = 55, *p* = 0.294, Fig. 6A). In patients showing a genetic response to aza/ven as illustrated above, we described a trend towards improved survival, which was not statistically significant (*p* = 0.183, Fig. 6B), possibly due to the relatively low number of patients. To assess the impact of functional BCL-2 dependence, we categorized patients as high versus low dependence on BCL-2, based on the individual BAD-HRK difference compared to the mean of the cohort. This analysis revealed a significantly longer overall survival in patients characterized by high versus low BCL-2 dependence (14 vs. 4.5 months; *p* = 0.038, Fig. 6C).

### Clonal evolution and acquired mechanisms of resistance to aza/ven

**Fig. 7:**
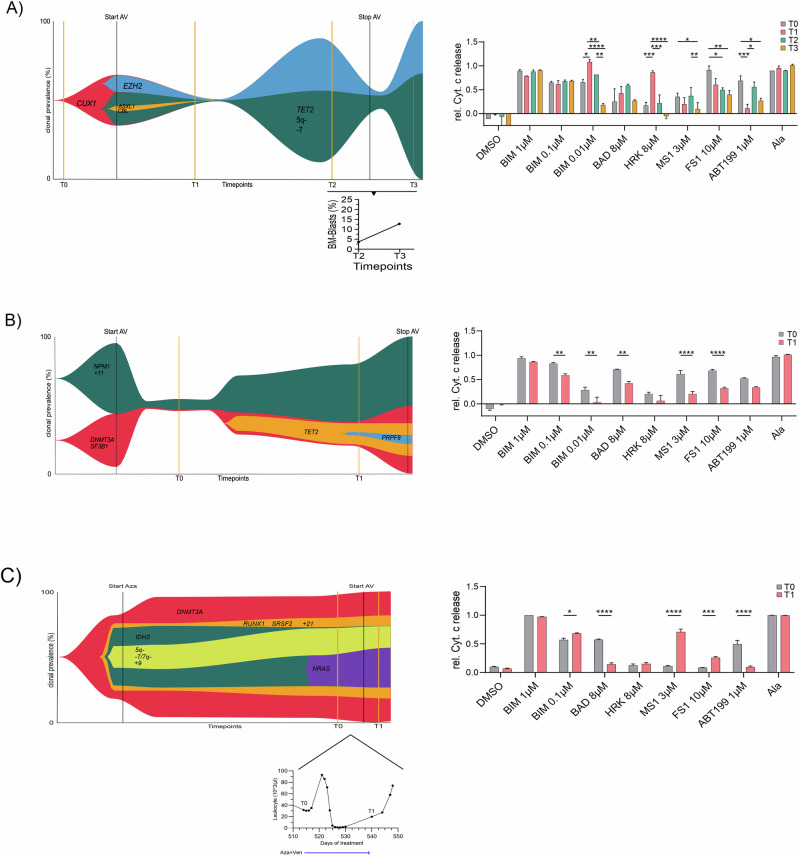
Clonal dynamics and BH3 profiling in three selected patients. **A**–**C** Fish plot analysis depicting treatment-associated clonal evolution in three different patients and their clinical course considering BM blast percentage (**A**) and Leukocyte counts (**C**). On the right, the corresponding longitudinal BH3 profiles of the same patients.

Figure 7 exemplarily presents fish plots illustrating the clonal dynamics and changes in apoptosis dependencies in three patients, highlighting functional mechanisms of apoptosis evasion and acquiring resistance to the aza/ven combination therapy. The first two patients (#44 and #53) are part of the current study cohort, whereas the third patient (Fig. 7C) originates from a previous project that served as preliminary work, providing foundational data to support and guide the current study.

In this case, the patient received 12 cycles of aza monotherapy prior aza/ven regimen, without significant changes in the clonal architecture. BH3 profiling was performed immediately before ven initiation, coinciding with the emergence of an *NRAS* mutation, and again after aza/ven treatment initiation. The observed switch in apoptotic dependencies likely reflects a ven-driven effect rather than changes induced by azacitidine alone. This interpretation is supported by in vitro BH3 profiling, where multiple cell lines showed increased BCL-xL dependence after ven exposure, highlighting BCL-xL upregulation as a specific adaptation to BCL-2 inhibition.

**Fig. 8:**
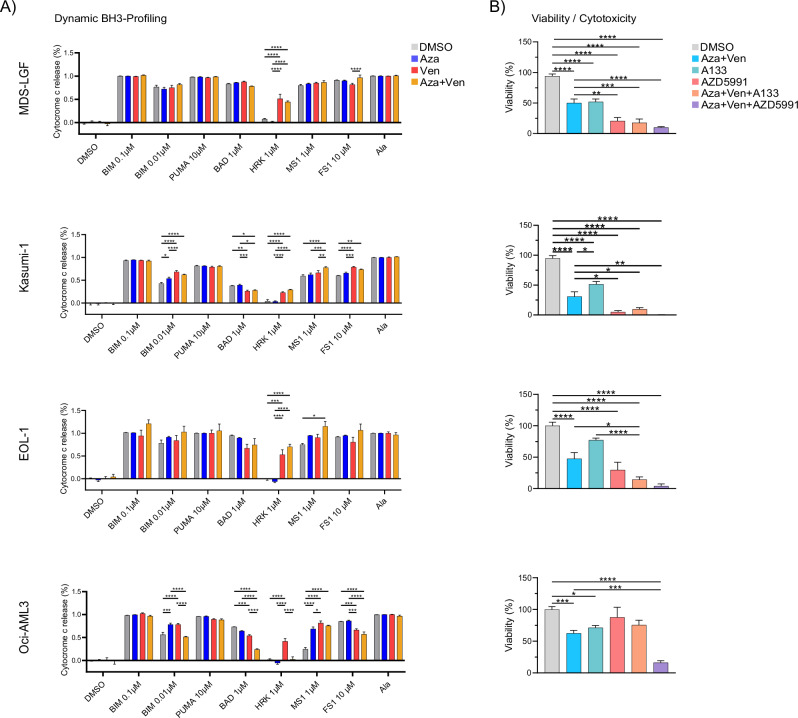
Dynamic BH3 profiling and viability assay in cell line models. **A** Dynamic BH3 profiling of cell lines treated with DMSO vs. aza vs. ven vs. aza/ven. **B** Corresponding cytotoxicity assays assessing the viability of cell lines treated with DMSO, aza/ven, the BCL-xL selective inhibitor A1331852 (A133), the selective MCL-1 inhibitor AZD5991, and combinations of aza/ven+A133 and aza/ven+AZD5991.

BH3 profiling revealed distinct mechanisms of acquired resistance to aza/ven associated with clinical disease progression: In Fig. 7A, early BH3 profiling during treatment shows a significant increase in BCL-xL dependence. By timepoint T3, BH3 profiling shows a broad loss of apoptotic priming, consistent with resistance to induction of apoptosis in general. This loss was associated with a marked expansion of genetic clone sizes and clinical disease progression. Similarly, in Fig. 7B, BH3 profiling identifies a broad decrease in apoptotic priming at the time of genetic progression, further underscoring the role of apoptotic evasion as a mechanism of resistance and supporter of genetic evolution. Finally, in Fig. 7C, BH3 profiling initially identifies a strong BCL-2 dependence. Indeed, the patient experienced a clinical (but not genetic) response to aza/ven with a rather stable clonal architecture until the emergence of the *NRAS* mutation. However, this was followed by rapid disease progression. At this timepoint, BH3 profiling revealed a functional shift from BCL-2 to MCL-1 dependence. The finding of differential BCL2-dependence and a potential role of BCL-xL and MCL-1 impacting sensitivity to AV prompted further mechanistic studies in cell lines. To this end, we employed established cell lines of MDS (MDS-LGF) and AML (Kasumi-1, EOL-1, Oci-AML3). To assess the impact of AV on mechanisms regulating mitochondrial apoptosis, we performed dynamic BH3 profiling in cells, treated with aza/ven for 72 h (Fig. 8A). Notably, BH3 profiling exhibited an increase in HRK-mediated cytochrome c release in MDS-LGF, Kasumi-1, and EOL-1 cells, but not in Oci-AML3. This finding suggests an important role of BCL-xL in preventing apoptosis in cells treated with aza/ven.

To assess the impact of aza/ven on mitochondrial apoptotic regulation, we performed dynamic BH3 profiling on AML and MDS cell lines after 72 h of treatment with aza, ven, or their combination (Fig. 8A). Notably, BH3 profiling revealed an increase in HRK-mediated cytochrome c release, indicative of BCL-xL dependence, in MDS-LGF, Kasumi-1, and EOL-1 cells following treatment with ven alone and aza/ven. Interestingly, in OCI-AML3, the increase in BCL-xL dependence was observed only with ven monotherapy, but not with the combination. These results suggest that BCL-xL plays a role in apoptotic adaptation following BCL-2 inhibition, and that this mechanism may arise independently of combination treatment with aza.

To further test this hypothesis, we performed drug combination tests and assessed the cytotoxic potential of aza/ven with and without the BCL-xL-specific inhibitor A1331852 (hereafter referred to as A133). In line with the results of dynamic BH3 profiling, A1331852 significantly increased the cytotoxic activity of aza/ven in MDS-LGF, Kasumi-1 and EOL-1, but not in Oci-AML3 (Fig. 8B). Together, this data further supports the functional role of BCL-xL as a targetable anti-apoptotic protein mitigating the therapeutic efficacy of aza/ven. Additionally, BH3 profiling revealed increased MCL-1 dependencies across the cell lines treated with aza/ven, as indicated by enhanced cyt c release in response to MS1 peptide. This finding aligns with the ex vivo treatment results, where the MCL-1 inhibitor AZD5991 exhibited comparable toxicity to A1331852 across most cell lines (Fig. 8B).

## Discussion

We here present a comprehensive mono-center analysis characterizing 55 MDS and AML patients treated with azacitidine ± venetoclax (aza±ven) on a clinical, genetic, proteomic and functional profiling level. Our study combines an in-depth analysis of genetic evolution along with insights into the regulation of the mitochondrial apoptosis pathway. This work is especially relevant in the context of MDS and AML treatment, as we aimed to identify potential biomarkers of response and resistance and to gain insight in the mechanisms behind acquired resistance to the combination of azacitidine and venetoclax (aza/ven) therapy in the context of clonal evolution. We discovered that sensitivity and resistance mechanisms are driven by differential functional dependencies on BCL-2 family members. Notably, our findings consistently show that (A) functional dependence on BCL-2 at initiation of treatment correlates with treatment response and prolonged or improved survival and (B) decreased mitochondrial apoptotic priming and functional shifts in anti-apoptotic dependencies are associated with acquired resistance to aza/ven.

However, results, particularly in subgroup analyses, should be interpreted with caution due to limited sample size and statistical power.

Furthermore, longitudinal genetic analyses revealed dynamic clonal shifts during treatment. Whole-exome sequencing was performed using tumor–normal pairs, with CD3⁺-enriched peripheral blood cells as germline controls. Variants were called using Mutect2, filtered against public databases, and sample concordance was >99% by Conpair analysis. Because orthogonal validation was not performed, some variants identified as somatic mutations may be sequencing artifacts, which represents a limitation of this study.

The combination aza/ven significantly improved patient outcomes in AML and also showed promising activity in hr-MDS (NCT02942290) [31–33]. Based on this, aza/ven versus azacitidine monotherapy is currently tested in a phase III trial (NCT04401748). The role of aza has been described in the literature as a synergistic inhibitor of the proteins MCL1 and BCL-xL, thereby increasing the dependence of leukemia cells on BCL2 [34, 35]. Indeed, the clinical efficacy of aza/ven is meaningful. However, biomarkers of response and resistance remain elusive. In AML, the European Leukemia Net (ELN) classification system poorly discriminates aza/ven outcomes and alternative patterns of mutations involving *TP53, FLT3* internal tandem duplication (*FLT3*-ITD), *KRAS* and *NRAS* mutations were suggested to categorize pts into higher-, intermediate-, and lower-benefit groups [36].

In our cohort, an analysis combining patients with *IDH1/2* or *FLT3* mutations revealed that 90% (9/10 patients) of evaluable patients treated with aza/ven responded, whereas only 33% (1/3 patients) treated with aza alone did. While limited in size, this trend is consistent with prior reports suggesting that *FLT3* and *IDH1/2* mutations may confer sensitivity to aza/ven-based regimens.

A key observation from our study was the heterogeneity in apoptotic priming within MDS/AML leukemic cell populations at diagnosis, which varied markedly in response to specific BH3 peptides targeting individual BCL-2 family members. Our results showed that high BCL-2 dependency at baseline, as indicated by BH3 profiling, strongly correlated with a favorable response to aza/ven therapy. This aligns with previous studies in AML and other malignancies [20, 37–40], where BCL-2 expression patterns predicted susceptibility to BH3 mimetics, such as ven. These findings highlight BCL-2 as a key player in determining the apoptotic threshold in MDS and AML. Furthermore, they support the notion that BCL-2 inhibition, in combination with hypomethylating agents, triggers metabolic and apoptotic crises in leukemic cells. This event involves alterations in the mitochondrial outer membrane permeabilization (MOMP) and the subsequent release of cytochrome c [41].

In our cohort of patients, we noted a potential role of BCL-xL in both primary and secondary resistance to aza/ven, as patients not achieving a genetic response to aza/ven showed an increased dependence on BCL-xL at initiation of treatment and longitudinal profiling also revealed an increase of BCL-xL dependence in response to aza/ven treatment (Figs. 7 and 8). A potential role of BCL-xL in this scenario was further supported by in vitro analyses also showing an increase of BCL-xL dependence in cell lines treated with aza/ven, together suggesting a mechanism of escaping cell death under aza/ven treatment. This mechanism was mechanistically validated by co-treatment with A1331852, a BCL-xL-specific inhibitor. This result indicates that BCL-xL upregulation may serve as an adaptive resistance mechanism in some cases, underscoring the need to evaluate BCL-xL levels when considering aza/ven therapy for hr-MDS patients. These insights support the growing interest in BCL-xL as a co-target for overcoming resistance in various malignancies treated with BCL-2 inhibitors.

The literature has already described the potential benefit of using BH3 profiling as a promising tool to identify patients likely to respond to therapy and discover mechanisms of acquired resistance [26, 42].

In contrast to ex vivo drug response assays such as those used in the VenEx trial [43], BH3 profiling directly measures mitochondrial apoptotic priming and functional anti-apoptotic protein dependence. This approach captures the intrinsic cell death threshold without requiring ex vivo drug treatment, and may provide a complementary tool for predicting ven response, particularly in heterogeneous or low-blast samples.

Notably, in relapsed/refractory (R/R) patients and those with genetic signatures associated with lower response rates, BH3 profiling might work as a promising biomarker with significant clinical utility. Our functional screening at diagnosis using BH3 profiling revealed heterogeneity, both in patient samples and in our preclinical MDS/AML cell line models. Yet, we observed a significant difference in BCL-xL dependencies between responders and non-responders. Moreover, in cases from our cohort of patients with clonal dynamics, we could observe differences in apoptotic priming together with alterations in protein expression. The analysis of the proteomic data, based on hallmark gene sets, comparing the entire cohort of patients (*n* = 12) to healthy controls (*n* = 3), revealed expected findings consistent with the literature. Notably, we observed the downregulation of oxidative phosphorylation (OXPHOS) and respiratory chain complex, alongside the upregulation of glycolysis-related pathways. These observations align with previous reports and reflect known metabolic adaptations in AML [41, 44].

We acknowledge that the relatively small size of our patient cohort represents a limitation of this study. As a result, our analyses primarily offer insights into biological processes involved in the pathogenesis of the disease, such as mitochondrial metabolism, rather than allowing for a robust stratification between responders and non-responders to draw clinically actionable conclusions regarding mechanisms of response to aza/ven therapy. Future metabolomics studies involving larger, well-annotated cohorts will be required.

Therapy resistance, not only in hematological malignancies but across various tumor types, has increasingly been attributed in part to the overexpression of anti-apoptotic Bcl-2 protein family members, particularly BCL-xL and MCL-1 [45, 46]. Despite the efforts to develop several compounds targeting these proteins, many have failed in the clinic settings due to on-target toxicities. In this study, we adopted an in vitro approach to confirm BH3 profiling results to investigate these mechanisms of resistance and show a heterogeneously increased susceptibility to selective inhibition of BCL-xL and/or MCL-1 in cell lines treated with aza/ven, suggesting targetable mechanisms of escape. To translate these findings into clinical concepts, targeted inhibitors of BCL-xL and MCL-1 are under development. For example, DT2216, a selective BCL-xL degrader, taking advantage of differential expression of Von Hippel Lindau E3 ligase [47], demonstrated antitumor activity with minimal effect on platelets providing hope for a reduction of thrombocytopenia in patients. Similarly, the dual BCL-2/BCL-xL inhibitor pelcitoclax, exploiting a prodrug strategy, has shown promising results in reducing thrombocytopenia while maintaining efficacy against metastatic solid tumors [48]. Another innovative agent, ABBV-155, an antibody-drug conjugate of the BCL-xL inhibitor clezutoclax, is currently under clinical evaluation [49, 50].

Certain hematological malignancies, such as AML and multiple myeloma, rely on MCL-1 for survival and may adopt the MCL-1 pathway to evade apoptosis in R/R contexts. Recent clinical trials have highlighted on-target cardiac toxicity as a significant limitation of MCL-1 inhibitors. Advances in proteolysis targeting chimera (PROTACs) technology appear to mitigate these critical side effects [51–53].

In summary, we here provide insights into treatment-associated clonal evolution in MDS and AML and propose functional profiling assays, such as BH3 profiling, to identify potential biomarkers and targetable mechanisms of resistance as a basis for optimized therapeutic strategies.

## Supplementary information

Supplementary Methods_Tables

Supplementary Figures

## Data Availability

For original data, please contact raphael.koch@med.uni-goettingen.de.
