# Supplementary material for: Clonal evolution and apoptosis resistance in myelodysplastic neoplasms and acute myeloid leukemia under treatment: insights from integrative longitudinal profiling
Source: Leukemia. 2025 Sep 19;39(12):3026–36. doi: 10.1038/s41375-025-02756-7 (PMC12634422; doi:10.1038/s41375-025-02756-7)
Supplement: Supplementary file 1 — Supplementary Methods_Tables [file 41375_2025_2756_MOESM1_ESM.docx]

**Supplementary Methods and Tables**

**Supplementary Methods**

*Patient cohort*

This monocenter study enrolled 55 consecutive patients mainly with myelodysplastic neoplasms (MDS) and acute myeloid leukemia (AML) diagnosed and treated at the University Medical Center Göttingen (UMG): 23 patients with MDS, 32 AML between 2019 and 2024. Patients were included if they had at least received one cycle of azacitidine ± venetoclax. At the time of enrollment, 20 patients were treated with aza monotherapy, while 35 received the combination of aza/ven. The median number of treatment cycles was 2.5 [range: 1–23] for aza monotherapy and 3 [range: 1–17] for aza/ven. Treatment data, including cycle counts for additional regimens, are summarized in Table 1.

The study was approved by the local ethic committees (application number 30/3/18) and supported by the Hector Foundation. Informed consent was obtained from all patients.

*CD34⁺ Cell Enrichment and Rationale*

CD34⁺ cells were immunomagnetically enriched from peripheral blood or bone marrow mononuclear cells before genetic analysis. This approach was implemented to ensure consistent sampling of hematopoietic stem and progenitor cells across all patients. CD34 expression was confirmed in all analyzed samples, supporting its reliability as a selection marker in both disease contexts. Additionally, in MDS cases, we routinely use CD34⁺ mononuclear cell enrichment, and previous work from our group has shown that peripheral blood–derived CD34⁺ cells yield comparable genetic results to those from bone marrow, facilitating longitudinal monitoring while minimizing patient discomfort.^1–5^

*Cell lines*

Human AML cell lines including OCI-AML3 (DSMZ No. ACC 582; FAB M4, sex male), Kasumi-1 (DSMZ No. ACC 220, FAB M2, sex male) and EOL-1 (DSMZ No. ACC 386, FAB not indicated, sex male) were obtained from DSMZ. MDS-LGF (RRID:CVCL_A8QZ, MDS, sex male) was obtained from Kaoru Tohyama (Kawasaki Medical School, Okayama, Japan). OCIAML3 and EOL-1 were cultured in RPMI 1640 (ThermoFisher, Cat. No. 11875093), supplemented with 20% FBS, 4 mM L-glutamine, 100 IU/ml penicillin and 100 mg/ml streptomycin. Kasumi-1 was cultured in Advanced RPMI (ThermoFisher, Cat. No. 12633012) supplemented with 5% FBS, 4 mM L-glutamine, 100 IU/ml penicillin and 100 mg/ml streptomycin. MDS-LGF was cultured in RPMI 1640, supplemented with 10% FBS, 4 mM L-glutamine, 100 IU/ml penicillin and 100 mg/ml streptomycin. All cell lines were kept at 37°C in a humidified 5% CO2 incubator. Cells were routinely tested for mycoplasma contamination by a polymerase chain reaction (PCR)-based assay (Venor^®^GeM Classic, Cat. No. 11-1025G).

*Genetic analysis*

All genetic abnormalities reported were somatic and non-constitutional. Chromosome banding analysis (CBA) of G-banded chromosomes from bone marrow cultures was performed as described previously. ^1,2,6^ Fluorescence in situ hybridization (FISH) analysis utilized a specific panel designed for MDS and AML patients, accounting for up to 18 probes. These probes are commercially available (Supplementary Table 1) and were used as previously described.^1,3,4^

Targeted next-generation sequencing (NGS) was performed in-house using the QIAseq Targeted DNA Panel (Qiagen), covering up to 53 genes relevant to myeloid neoplasms (Supplementary Table 2). This technology employs a primer extension–based enrichment strategy, in which gene-specific primers anneal to target regions, followed by extension and amplification before library preparation. Libraries were sequenced on an Illumina MiniSeq platform according to the manufacturer's instructions.

FISH and sequencing were done either on bone marrow or peripheral blood CD34+ cells after immunomagnetic enrichment. Whole exome sequencing (WES) was performed on gDNA extracted from bone marrow or peripheral blood cells after CD34+ immunomagnetic enrichment in patients with clinical progression but no detectable clonal evolution. WES was carried out with Roche NimbleGen SeqCap EZ Exome V3/Agilent SureSelect Human All Exon V6 exon capture kits. Preliminary quantification of WES libraries using Qubit 3.0, coupled with Agilent 2100 analysis was used to determine insert size. The concentration of the libraries (library concentration > 3nM) was quantified by qPCR (Supplementary Methods). Whole exome sequencing (WES) was performed at Biomarker Technologies (BMK) GmbH (Münster, Germany) on an Illumina Novaseq 6000. Preliminary downstream analysis on the WES data using an in-house analysis pipeline by BMK GmbH was provided together with the sequencing service. Germline variants were excluded using standard filtering approaches for both targeted sequencing (based on a customized panel of up to 53 genes) and whole-exome sequencing (WES). Specifically, variants were filtered against population databases such as gnomAD, HaplotypeCaller tool from the GATK software, 1000 Genomes, and ExAC, excluding those with a minor allele frequency (MAF) >1% in any population. Common polymorphisms and variants present in matched normal controls were also removed. For WES, genomic DNA from tumor cells and matched CD3+ T cells (enriched from peripheral blood) was used to distinguish somatic from germline variants through paired analysis. Only rare, non-synonymous, and high-confidence somatic variants were retained for downstream analysis. The most common rearrangements in MDS/AML were screened by adopting the cDNA-based, CE-IVD kit, Mentype® AMLplex^QS^ able to detect 11 gene fusions and 34 transcript variants.

To define genetic response to aza/ven, two criteria were applied: at least a 50% decrease in clone size by FISH and/or chromosome banding analysis according to the Cheson criteria^7^ and/or a decrease of at least 10% or more in variant allele frequency (VAF).^8^ Additionally, statistical comparison of paired pre- and post-treatment of clone size was performed using a paired t-test, and only statistically significant changes (p < 0.05) were considered evidence of genetic response, particularly in case with multiple genetic aberrations.

*Whole Exome Sequencing*

Prior to further analysis, data quality necessitates filtration. Subsequent to filtration, high-quality data is aligned against a reference genome sequence. The software and methodologies employed in this process align with the prevailing techniques and tools within the domain of human disease research. Specifically, the BWA mem^9^ method is used for alignment, while the mutect2^10^ software is employed for somatic mutation detection, and the Control-FreeC^11^ software facilitates the detection of somatic copy number variation (CNV).

These were then used as input for GISTIC2.0 to assess CNV distribution and reproducibility across samples.^12^ The CNV level was determined based on the t-value, which represents the copy number change. The cutoffs classify deviations into no, low, or high levels of amplification or deletion based on the value of t. The default threshold values for copy number amplifications/deletions were assessed using GISTIC2.0.

Amplification: no amplification with t<0.1; low-level with t between 0.1-0.9 and high-level with t>0.9.

Deletion: no amplification with t>-0.1; low-level with t between -1.3 and -0.1 and high-level with t< -1.3.

The clean reads of the sample are aligned to the reference genome using the BWA software, resulting in the initial alignment output in BAM format (http://SAMtools.github.io/hts-specs/SAMv1.pdf). The SortSam tool from Picard^13^ software is utilized to sort the alignment results, followed by the MarkDuplicates tool for duplicate marking.

*Copy number analysis*

The GATK^14^ software was used for calling copy number aberrations. GATK was employed to perform Base Quality Score Recalibration (BQSR) on the duplicate-marked BAM file. Finally, statistics related to coverage and depth were computed on the alignment results.

Single nucleotide variation (SNV) and insertion/deletion (Indel) detection employs the mutect2^10^ toolkit within the GATK software package.^14^ Preprocessing involves utilizing Picard for deduplication (MarkDuplicates) and base quality recalibration based on the alignment outcomes of the clean reads against the reference genome. This safeguards the accuracy of detecting somatic SNVs and Indels. For detecting Somatic CNVs and B allele frequency (BAF) on SNV sites, Control-FREEC^11^ (http://bioinfo-out.curie.fr/projects/freec/) software was adopted. This tool accommodates analysis of super-tetraploid tumor samples and tumors mixed with normal cells.

Copy number variants were classified based on t-values indicating the degree of copy number deviation: high-level amplifications were defined as t>0.9, low-level amplifications as t=0.1-0.9, and deletions were categorized analogously, with high-level deletions defined as t< -1.3 (Supplementary Figure 3).

*BH3 profiling on primary patient samples*

Peripheral blood mononuclear cells (PBMCs) and bone marrow mononuclear cells (BMMCs) were isolated using ficoll gradient centrifugation according to standard protocols. PBMCs and BMMCs samples were stained with anti-human CD34 (dye: PE-Cy5. Beckman coulter, Product No:IM2650U), anti-human CD45 (dye: FITC. Biolegend, clone HI-30. cat #982316) fluorescently labeled antibodies for flow cytometry. Next, functional BH3 profiling was performed.^15–17^ The flow cytometry gating strategy is depicted in Supplementary Figure 5. Brieﬂy, cells were exposed to several concentrations of synthetic BH3 peptides in MEB buffer (150mM mannitol, 10mM HEPES-KOH pH 7.5, 50mM KCl, 0.02mM EGTA, 0.02mM EDTA, 0.1% BSA and 5mM Succinate) after plasma membrane permeabilization with 0.002% digitonin. Dimethylsulfoxide (DMSO) is an organosulfur compound used as a negative control for cytochrome c retention and Alamethicin (Ala) is a peptide-antibiotic that induces pores in the mitochondrial membrane and thus serves as a positive control for cytochrome c release. The BIM peptide activates the pro-apoptotic effector proteins BAX and BAK and thus reveals a cell's capability of undergoing apoptosis. BAD antagonizes BCL-2, BCL-xL and BCL-w. HRK specifically antagonizes BCL-xL. MS1 specifically antagonizes MCL-1. FS1 specifically antagonizes BFL1. PUMA is a pan-sensitizer (as well as contributing to activating BAX and BAK) and through its BH3 domain neutralizes antiapoptotic Bcl-2 members. Cells were incubated with the BH3 peptides for 1 hour at 25°C and subsequently ﬁxed with 4% paraformaldehyde for 10 minutes, followed by neutralization for 10 minutes using N2 buffer (1.7M Tris, 1.25M Glycine pH 9.1). Finally, intracellular cytochrome c (cyt c) was stained with an immunoﬂuorescence-labeled antibody (dye: Alexa fluor. Biolegend, clone 6H2.B4 cat. # 612310) and cells were subjected to ﬂow cytometry (LSRFortessa X-20, Becton Dickinson) to assess cyt c release in the patients’ MDS/AML blast cells (defined by CD45 lo-mid/SSC-low and CD34 expression). Relative cyt c release in each population was assessed by 1-[(sample-pos.ctrl.)/(neg.ctrl.-pos.ctrl.)].

*In vitro* cytotoxicity assays

For cytotoxicity assays with cell lines, cell lines were seeded at 25.000 cells per well in 96-well plates and treated with Aza 1µM, Ven 100nM, A1331852 (BCL-xL selective inhibitor; hereafter referred to as A133) 100nM, AZD5991 (Mcl-1 specific inhibitor) 100nM for 72h. DMSO 0.1% was adopted as a negative control. Cells were subjected to SpectraMax M (Molecular Devices) to assess cell viability as an end-point assay after staining with alamarBlue™ (ThermoFisher).

*Statistics*

Statistical analysis was performed using Prism 10.1.2 (GraphPad Software, LCC) and R.^18^ Patient characteristics were compared using Fisher´s exact test for categorical variables and the Wilcoxon rank-sum test for continuous variables. OS was calculated from treatment initiation with aza/ven to death from any cause, with censoring at the last follow-up and at allogeneic stem cell transplantation (Allo-SCT). All reported p-values are nominal and unadjusted for multiple comparisons. Given the hypothesis-driven nature of the analyses and the predefined comparisons based on biological rationale, formal correction for multiple testing was not applied.

To explore biological pathways associated with differential protein expression, Gene Set Enrichment Analysis (GSEA) was performed using the clusterProfiler R package.^19^ Proteins were ranked based on the product of the negative log-transformed adjusted p-values and the sign of the log2 fold-change. The enrichment analysis was conducted against the Molecular Signatures Database (MSigDB) hallmark gene sets.^20^ Normalized enrichment scores (NES) and adjusted p-values (false discovery rate, FDR) were calculated to determine statistical significance. Significant pathways were defined using an adjusted p-value threshold of <0.05.

Two-way ANOVA and Bonferroni post-test were performed both for BH3 profiling and one-way ANOVA and Bonferroni post-test for cell lines ex-vivo treatment. Data are presented as mean ± standard error of the mean (SEM) unless stated otherwise. *p*-values were annotated using APA (American Psychological Association) style as follows: *p* *≤* 0.05 (*), p *≤* 0.01 (**) and *p ≤* 0.001 (***). Data visualization was performed using the packages maftools v2.10.05,^21^ clusterProfiler v4.2.2,^19^ IPSSM v1.0.0,^22^ fishplot v0.5.1,^23^ ggpubr v.0.4.0^24^ and ggplot2 v3.5.1^25^ in R.^18^

*Proteomics*

Proteomics was performed on protein lysate from either bone marrow or peripheral blood CD34+ cells after immunomagnetic enrichment. The cells were lysed by suspension in Urea buffer (8M Urea, 20mM HEPES pH 8.0, 1mM sodium orthovanadate, 2.5mM sodium pyrophosphate, 1mM beta-glycerophosphate), followed by 3 cycles of sonication. After protein concentration determination in the cleared lysate using the Pierce 660 nm assay (Thermo Fisher Scientific) the samples were reduced with 5 mM DTT for 1h at 37°C and alkylated with 10 mM iodoacetamide for 30 min at room temperature and in the dark. The samples were incubated with Lys-C (Wako) for 2 h at 37 °C (enzyme-to-substrate ratio of 1:50) and after dilution to 1 M urea with 20 mM HEPES pH 8.0 proteolysis was continued overnight with trypsin (Promega) in an enzyme-to-substrate ratio of 1:50. Afterwards, the samples were purified using C18 spin columns (Harvard Apparatus), dried by vacuum centrifugation and dissolved in 50 mM triethylammonium bicarbonate (TEAB). Peptide concentration was determined using Pierce Fluorometric Assay (Thermo Fisher Scientific) and for protein quantitation, the samples were labeled with tandem mass tags (TMT) according to the instructions of the manufacturer (Thermo Fisher Scientific). Briefly, 5 µg of peptides per sample were labeled with 50 µg of TMT 11-plex reagents for 1 h at room temperature and the reactions were quenched by incubation for 15 min with hydroxylamine in a final concentration of 0.2 %. The individually labeled samples were mixed and the combined sample was dried by vacuum centrifugation. Each multiplex contained 5 µg of an internal reference that was prepared by mixing equal peptide amounts of each sample and TMT labeling. The multiplexed samples were dried by vacuum centrifugation and separated into 8 fractions using Pierce High pH RP fractionation kit (Thermo Fisher Scientific). For LC/MS the samples were vacuum dried, dissolved in 0.1% FA and analyzed on an Ultimate 3000 nRSLC nano-UHPLC system coupled to a Q Exactive HF quadrupole-Orbitrap hybrid mass spectrometer via a Nanospray Flex nano-ESI ion source (all Thermo Fisher Scientific). After desalting on a C18 trap column (5 cm, packed with ReproSil-Pur 120 C18-AQ, 5µm particle size, Dr. Maisch GmbH), the samples were separated on a C18 analytical column (32 cm, packed with ReproSil-Pur 120 C18-AQ, 1.9µm particle size, Dr. Maisch GmbH) in a 118 min method with a linear gradient of 2 to 40% solvent B (80% ACN, 0.1% FA) at a flow rate of 300 nl/min. The eluted peptides were ionized and analyzed in tandem MS (MS/MS) experiments by data-dependent acquisition. Survey scans of the ion population were acquired with the Orbitrap analyzer in the range of m/z 350-1600 with a resolution setting of 120,000 FWHM at m/z 200 and including charge states 2-6. The 20 most abundant precursor ions were selected for collision-induced dissociation (HCD) with normalized collision energy of 32 % using an isolation window of m/z 1.4. Fragment ion spectra were acquired in the Orbitrap with a resolution setting with a resolution setting of 60,000 FWHM at m/z 200 fixed first mass of m/z 110. AGC target values and maximum injection times for MS and MS/MS were set to 1x10^6 in 40 ms and 5x10^4 in 90 ms, respectively. Already fragmented precursor ions were excluded from repeated isolation for 20 s.

The raw data was processed with version 2.1.4.0 of the MaxQuant software (PMID 19029910) by matching the mass spectra against the Uniprot human reference proteome (Swiss-Prot/TrEMBL, release 03-2023) and a collection of frequently observed laboratory contaminants using the integrated Andromeda peptide search engine. The mass tolerances were set to 4.5 ppm and 20 ppm for precursor and fragment ions, respectively. Oxidation of methionine and acetylation of the protein N-terminus were defined as variable modifications and carbamidomethylation of cysteine was considered as fixed modification. The minimal peptide length was set to seven amino acids and maximum two missed cleavage sites for proteolysis were tolerated. Both on the peptide and protein level the FDR was set to 0.01 using a decoy approach by searching the reversed database. The search engine output was filtered by removing potential contaminants, hits to the decoy database and proteins identified solely based on modified peptides. The TMT reporter ion intensities were adjusted for equal sample loading within multiplexes by standardization on the median summed-up values of each labeling channel and scaled between the multiplexed samples according to the internal reference.

**References**

1. Braulke F, Platzbecker U, Müller-Thomas C, et al. Validation of cytogenetic risk groups according to International Prognostic Scoring Systems by peripheral blood CD34+FISH: results from a German diagnostic study in comparison with an international control group. *Haematologica*. 2015;100(2):205-213. doi:10.3324/HAEMATOL.2014.110452

2. Haase D, Feuring-Buske M, Konemann S, et al. Evidence for malignant transformation in acute myeloid leukemia at the level of early hematopoietic stem cells by cytogenetic analysis of CD34+ subpopulations. *Blood*. 1995;86(8):2906-2912. doi:10.1182/blood.v86.8.2906.bloodjournal8682906

3. Braulke F, Schanz J, Jung K, et al. FISH analysis of circulating CD34+ cells as a new tool for genetic monitoring in MDS: verification of the method and application to 27 MDS patients. *Leuk Res*. 2010;34(10):1296-1301. doi:10.1016/J.LEUKRES.2010.01.010

4. Braulke F, Jung K, Schanz J, et al. Molecular cytogenetic monitoring from CD34+ peripheral blood cells in myelodysplastic syndromes: First results from a prospective multicenter German diagnostic study. *Leuk Res*. 2013;37(8):900-906. doi:10.1016/J.LEUKRES.2013.03.019

5. Martin R, Acha P, Ganster C, et al. Targeted deep sequencing of CD34+ cells from peripheral blood can reproduce bone marrow molecular profile in myelodysplastic syndromes. *Am J Hematol*. 2018;93(6):E152-E154. doi:10.1002/AJH.25089

6. Haase D, Germing U, Schanz J, et al. New insights into the prognostic impact of the karyotype in MDS and correlation with subtypes: Evidence from a core dataset of 2124 patients. *Blood*. 2007;110(13):4385-4395. doi:10.1182/blood-2007-03-082404

7. Cheson BD, Greenberg PL, Bennett JM, et al. Clinical application and proposal for modification of the International Working Group (IWG) response criteria in myelodysplasia. *Blood*. 2006;108(2):419-425. doi:10.1182/blood-2005-10-4149

8. Mazzeo P, Ganster C, Wiedenhöft J, et al. Comprehensive sequential genetic analysis delineating frequency, patterns, and prognostic impact of genomic dynamics in a real-world cohort of patients with lower-risk MDS. *Hemasphere*. 2024;8(9):e70014. doi:10.1002/HEM3.70014

9. Li H, Durbin R. Fast and accurate short read alignment with Burrows–Wheeler transform. *Bioinformatics*. 2009;25(14):1754-1760. doi:10.1093/BIOINFORMATICS/BTP324

10. Cibulskis K, Lawrence MS, Carter SL, et al. Sensitive detection of somatic point mutations in impure and heterogeneous cancer samples. *Nature Biotechnology 2013 31:3*. 2013;31(3):213-219. doi:10.1038/nbt.2514

11. Boeva V, Popova T, Bleakley K, et al. Control-FREEC: a tool for assessing copy number and allelic content using next-generation sequencing data. *Bioinformatics*. 2012;28(3):423-425. doi:10.1093/BIOINFORMATICS/BTR670

12. Mermel CH, Schumacher SE, Hill B, Meyerson ML, Beroukhim R, Getz G. GISTIC2.0 facilitates sensitive and confident localization of the targets of focal somatic copy-number alteration in human cancers. *Genome Biol*. 2011;12(4):1-14. doi:10.1186/GB-2011-12-4-R41/FIGURES/7

13. GitHub - broadinstitute/picard: A set of command line tools (in Java) for manipulating high-throughput sequencing (HTS) data and formats such as SAM/BAM/CRAM and VCF. https://github.com/broadinstitute/picard. Accessed September 17, 2024.

14. Depristo MA, Banks E, Poplin R, et al. A framework for variation discovery and genotyping using next-generation DNA sequencing data. *Nature Genetics 2011 43:5*. 2011;43(5):491-498. doi:10.1038/ng.806

15. Montero J, Letai A. Dynamic BH3 profiling-poking cancer cells with a stick. *Mol Cell Oncol*. 2016;3(3). doi:10.1080/23723556.2015.1040144

16. Ryan J, Montero J, Rocco J, Letai A. iBH3: simple, fixable BH3 profiling to determine apoptotic priming in primary tissue by flow cytometry. *Biol Chem*. 2016;397(7):671-678. doi:10.1515/HSZ-2016-0107

17. Koch R, Christie AL, Crombie JL, et al. Biomarker-driven strategy for MCL1 inhibition in T-cell lymphomas. *Blood*. 2019;133(6):566-575. doi:10.1182/BLOOD-2018-07-865527

18. Team RC. R: A Language and Environment for Statistical Computing. *R Foundation for Statistical Computing*. 2021.

19. Yu G, Wang LG, Han Y, He QY. clusterProfiler: an R package for comparing biological themes among gene clusters. *OMICS*. 2012;16(5):284-287. doi:10.1089/OMI.2011.0118

20. Subramanian A, Tamayo P, Mootha VK, et al. Gene set enrichment analysis: A knowledge-based approach for interpreting genome-wide expression profiles. *Proc Natl Acad Sci U S A*. 2005;102(43):15545-15550. doi:10.1073/PNAS.0506580102/SUPPL_FILE/06580FIG7.JPG

21. Mayakonda A, Lin DC, Assenov Y, Plass C, Koeffler HP. Maftools: efficient and comprehensive analysis of somatic variants in cancer. *Genome Res*. 2018;28(11):1747-1756. doi:10.1101/GR.239244.118

22. Bernard E, Tuechler H, Greenberg PL, et al. Molecular International Prognostic Scoring System for Myelodysplastic Syndromes. *NEJM Evidence*. 2022;1(7). doi:10.1056/EVIDOA2200008

23. Miller CA, McMichael J, Dang HX, et al. Visualizing tumor evolution with the fishplot package for R. *BMC Genomics*. 2016;17(1):880. doi:10.1186/s12864-016-3195-z

24. Kassambara A. ggpubr: “ggplot2” Based Publication Ready Plots. *CRAN: Contributed Packages*. July 2016. doi:10.32614/CRAN.package.ggpubr

25. Wickham H. ggplot2: Elegant Graphics for Data Analysis. *https://ggplot2.tidyverse.org*. 2016.

Supplementary Table 1. FISH probes included in the FISH panel.

| **Chromosome** | **Probe** | **Chromosome** | **Probe** |
| --- | --- | --- | --- |
| 1 | CDKN2C/CKS1B (1p32/q21) | 11 | *KMT2A* (*MLL*) (11q23) |
| 3 | MECOM (EVI1)-Rearr. (3q26) | 11 | NUP98-Rearrangement (11p15) |
| 4 | *TET2* (4q24) | 12/21 | *ETV6/RUNX1* (12p13/21q22) |
| 5 | EGR1/D5S23/D5S721 (5q31) | 13 | DLEU/LAMP(13q14/13q34) |
| 6,9 | DEK::NIP214/t(6;9)(p23;q34) | 15,17 | *PML::RARa* t(15;17)(q24;q21) |
| 7 | D7S522 (7q31)/CEP7 | 16 | *CBFB::MYH11* inv16(p13q22) |
| 8 | CEP 8 | 17 | *TP53/NF1* (17p13/17q11) |
| 8,20 | CEP8/D20S108 (20q12)/20qter(20q13) | 20 | D20S108 (20q12) |
| 8,21 | *RUNX1::RUNX1T1* t(8;21)(q22;q22) | X/Y | CEP X/Y |

Supplementary Table 2. Genes included in the 53 gene targeted NGS panel.

| **Gen** | **Transkript** | **Exon(s)** | **Gen** | **Transkript** | **Exon(s)** | **Gen** | **Transkript** | **Exon(s)** |
| --- | --- | --- | --- | --- | --- | --- | --- | --- |
| ***ABL1*** | NM_005157 | 4-6 | ***JAK3*** | NM_000215 | 13 | ***IDH2*** | NM_002168 | 4-6 |
| ***ASXL1*** | NM_015338 | 13 | ***KIT*** | NM_000222 | 2, 8-11, 13-15, 17-18 | ***IKZF1*** | NM_006060 | 2-8 |
| ***BCOR*** | NM_017745, NM_001123385 | 2-15 | ***KMT2A*** | NM_005933 | 1, 5-8 | ***JAK2*** | NM_004972 | 12, 14 |
| ***BCORL1*** | NM_021946 | 1-12 | ***KRAS*** | NM_033360 | 2-3 | ***WT1*** | NM_024424 | 7, 9 |
| ***BRAF*** | NM_004333 | 15 | ***MPL*** | NM_005373 | 10 | ***ZRSR2*** | NM_005089 | 1-11 |
| ***CALR*** | NM_004343 | 9 | ***MYD88*** | NM_001172566 | 3-5 |  |  |  |
| ***CBL*** | NM_005188 | 8-9 | ***NF1*** | NM_001042492,  NM_000267,  NM_001128147 | 1-58 |  |  |  |
| ***CDKN2A*** | NM_058195, NM_001195132 | 1-3 | ***NOTCH1*** | NM_017617 | 26-28, 34 |  |  |  |
| ***CEBPA*** | NM_004364, NM_001287424 | 1 | ***NPM1*** | NM_002520 | 11 |  |  |  |
| ***CSF3R*** | NM_000760, NM_172313 | 14-17 | ***NRAS*** | NM_002524 | 2-3 |  |  |  |
| ***CUX1*** | NM_001913, NM_181552 | 1-24 | ***PDGFRA*** | NM_006206 | 12, 14, 18 |  |  |  |
| ***DDX41*** | NM_016222 | 1-17 | ***PHF6*** | NM_032335, NM_032458 | 2-10 |  |  |  |
| ***DNMT3A*** | NM_022552, NM_153759 | 1-19 | ***PPM1D*** | NM_003620 | 1-6 |  |  |  |
| ***EPOR*** | NM_000121 | 8 | ***PRPF8*** | NM_006445 | 1-43 |  |  |  |
| ***ETNK1*** | NM_018638 | 3 | ***PTPN11*** | NM_002834 | 3, 13 |  |  |  |
| ***ETV6*** | NM_001987 | 1-8 | ***RAD21*** | NM_006265 | 2-14 |  |  |  |
| ***EZH2*** | NM_004456 | 2-20 | ***RUNX1*** | NM_001754, NM_001122607 | 1-9 |  |  |  |
| ***FLT3*** | NM_004119 | 14,15, 20 | ***SETBP1*** | NM_015559 | 4 |  |  |  |
| ***GATA1*** | NM_002049 | 2 | ***SF3B1*** | NM_012433 | 13-16 |  |  |  |
| ***GATA2*** | NM_032638 | 2-6 | ***SRSF2*** | NM_003016 | 1 |  |  |  |
| ***GNAS*** | NM_000516 | 8-9 | ***STAG2*** | NM_006603 | 2-33 |  |  |  |
| ***GNB1*** | NM_002074, NM_001282539 | 1-12 | ***TET2*** | NM_001127208 | 3-11 |  |  |  |
| ***HRAS*** | NM_005343 | 2-3 | ***TP53*** | NM_000546, NM_001126114 | 2-11 |  |  |  |
| ***IDH1*** | NM_005896 | 4-6 | ***U2AF1*** | NM_006758 | 2, 6 |  |  |  |

Supplementary Table 3. Univariate and multivariate Cox proportional hazards models.

| **Overall survival** | **Category** | **HR (univariable)** | **HR (multivariable)** |
| --- | --- | --- | --- |
| Gender | Male | - | - |
|  | Female | 1.03 (0.50-2.14, *p*=0.929) | - |
| Age | Continuous variable | *p*=0.393 | - |
| Disease_type | AML | - | - |
|  | MDS | 0.66 (0.31-1.39, *p*=0.269) | - |
|  | other | *p*=0.600 | - |
| Hemoglobin (g/dl) | Continuous variable | *p*=0.909 | - |
| Platelets (10^9^/L) | Continuous variable | *p*=0.372 | 0.98 (0.95-1.01, p=0.232) |
| Leukocyte (10^9^/L) | Continuous variable | *p*=0.460 | - |
| Neutrophil (%) | Continuous variable | *p*=0.956 | - |
| ANC (10^9^/L) | Continuous variable | *p*=0.499 | - |
| Bone marrow Blasts (%) | Continuous variable | *p*=0.423 | 1.04 (1.01-1.07, p=0.024)* |
| Peripheral blood blasts (%) | Continuous variable | *p*=0.190 | - |
| Numer of cycles | Continuous variable | - | - |
| Complex karyotype at recruitment | No | 1.14 (0.37-3.50, *p*=0.816) | - |
|  | Yes | - | - |
| Genetic response under aza/ven treatment | No | 0.37 (0.11-1.26, *p*=0.113) | 4.59 (0.67-37.41, p=0.120) |
|  | Yes | *p*=0.029* | 6.30 (0.04-889.29, p=0.466) |
| Bcl-2 dependencies prior initiation therapy | Continuous variable | - | - |
| Mean Bcl-2 dependencies prior initiation therapy | Below | 0.16 (0.04-0.64, *p*=0.008)* | 0.03 (0.00-0.44, p=0.012)* |
|  | Above | - | - |

AML, acute myeloid leukemia, MDS, myelodysplastic neoplasms, other (myeloproliferative neoplasm (MPN), MDN/MPN and chronic myelomonocytic leukemia (CMML); g, gram; dl, deciliter; platelets, L, liter; ANC (absolute neutrophils count). Hb, platelets, leucocytes, neutrophils, ANC, peripheral and bone marrow blasts count are values at diagnosis. Age, hb, platelets, leucocytes, neutrophils, ANC, PB and BM blasts and number of cycles have been considered in the analysis as continuous variables; aza/ven, azacitidine in combination with venetoclax.

Supplementary Table 4. Aberrations detected by chromosomal banding analysis and sequencing at diagnosis and at CE event during the course of the disease. CE, clonal evolution; CE indicates whether new mutations or cytogenetic abnormalities were detected between baseline and follow-up samples.

| **Pt.ID** | **Karyotype** | **Genes mutated** | **VAF (%)** |
| --- | --- | --- | --- |
| **2** | 47,XY,+14[11]/46,XY[9] | *U2AF1* NM_006758:exon6:c.470A>C:p.Q157P  *GATA2* NM_032638:exon6:c.1168_1170del:p.390_390del  *GATA2* NM_032638:exon3:c.599dupG:p.G200fs  *ASXL1* NM_015338:exon12:c.1927dupG:p.G642fs  *PHF6* NM_032335:exon2:c.66_67del:p.S22fs | 42.5  25.4  40.6  30.6  62.4 |
| **3** | 46,XY,t(8;11)(q24;q23),del(19)(p13.3)[12]/46,XY[1] | *KMT2A*-PTD |  |
| CE |  | *GATA2* NM_032638:exon5:c.1081C>T.R361C | 10.0 |
| **4** | 45,XY,der(1)del(1)(p31)t(1;20)(q31;q11)t(20;13)(q13;q13),del(7)(q21q35),der(12)t(12;?20)(p12;p?11),del(13)(q1?3),  der(14)t(1;14)(p36;p11),20,der(21)t(1;21)(p36;p11),der(21)t(1;21)(p31;p11)[1]/45,idem,der(1)inv(1)(p35q32)ins(1;2)(p32;q?31q?37)[1]/46,XY[1] | Negative |  |
| CE | Trisomy 8 in FISH |  |  |
| **5** | 46,XX[19]/46,XY[3] | *PHF6* NM_032335:exon8:c.823C>T:p.R275*  *RUNX1* NM_001122607:exon2:c.295dupG:p.D99fs | 29.0  24.4 |
| **6** | 48,XX,del(1)(p13p31),+del(1)(p13p31),del(5)(q12q33),+8[19]/46,XX[1] | *IDH2* NM_002168:exon4:c.419G>A:p.R140Q | 8.0 |
| **10** | 46,XY,del(20)(q11q13)[14]/46,XY[6] | *RUNX1* NM_001754:exon8:c.958C>T:p.R320* *SF3B1* NM_012433:exon14:c.1998G>T:p.K666N | 26.8  23.0 |
| CE | 46,XY,del(20)(q11q13)[15]/46,idem,del(12)(p11p13)[3]/47,idem,+8[2] | *BCOR* NM_017745:exon12:c.4621dupA:p.M1541fs  *RAD21* NM_006265:exon14:c.1734dupT:p.I579fs  *CUX1* NM_181552:exon24:c.4325G>C:p.S1442T  *WT1* NM_024424:exon7:c.1140dupG:p.S381fs | 83.4  46.1  6.2  5.1 |
| **11** | 46,XX[27] | *FLT3*-ITD^high^  *NPM1* NM_002520:exon11:c.859_860insTCTG:p.L287fs (typA) | Ratio:0.56  n.a |
| **12** | 46,XX[20] | *CUX1* NM_181552:exon23:c.3781C>T:p.R1261*  *TET2* NM_001127208:exon3:c.1630C>T:p.R544*  *SF3B1* NM_012433:exon15:c.2098A>G:p.K700E | 36.8  66.0  34.9 |
| CE |  | *CEBPA* NM_004364:exon1:c.388G>A:p.G130S  *CEBPA* NM_004364:exon1:c.338C>T:p.A113V | 6.2  8.3 |
| **13** | 46,XY,t(5;10)(q31;q2?3)[9]/46,XY[12] | *IDH1* NM_005896:exon4:c.395G>A:p.R132H  *JAK2* NM_004972:exon14:c.1849G>T:p.V617F  *TET2* NM_001127208:exon11:c.5686A>T:p.R1896W  *SF3B1* NM_012433:exon15:c.2098A>G:p.K700E | 36.1  57.8  6.0  27.2 |
| **15** | 46,XY[25] | *ASXL1* NM_015338:exon12:c.1927dupG:p.G642fs  *BCOR* NM_017745:exon4:c.287_288del:p.L96fs  *IDH2* NM_002168:exon4:c.419G>A:p.R140Q  *SRSF2* NM_003016:exon1:c.284C>A:p.P95H  *STAG2* NM_006603:exon16:c.1542_1545del:p.T514fs | 22.7  24.5  23.3  34.3  52.2 |
| **16** | 46,XX[7] | *BCOR* NM_017745:exon15:c.5044_5050del:p.F1682fs  *CUX1* NM_001913:exon8:c.647dupA:p.E216fs  *PHF6* NM_032335:exon8:c.789_790insGG:p.D263fs  *PHF6* NM_032335:exon8:c.790T>G:p.F264V  *SRSF2* NM_003016:exon1:c.284C>A:p.P95H  *STAG2* NM_006603:exon28:c.3019_3020insAGGC:p.S1007_S1008delins* | 17.2  17.8  11.4  11.3  19.3  18.8 |
| **17** | 47<2n>,XY,der(2)t(2;5)(p22;q23)t(5;14)(q35;q32),del(5)(q12),r(7)(p22q22),+8,t(8;18)(p12;q1?2),der(14)t(14;5)(q32;q12)  t(5;2)(q23;p22),der(20)t(20;21)(q12;q11.2),del(21)(q11.2)[18]/47,idem,t(1;5;17;6)(q21;q3?3;q21;p2?4)[5] | Negative |  |
| **18** | not available | Negative |  |
| **19** | 46,XX,del(5)(q14q34)[12]/46,XX[8] | *TET2* NM_001127208:exon10:c.4479delG:p.K1493fs | 20.5 |
| CE |  | *TP53* NM_000546:exon7:c.733G>A:p.G245S  *TP53* NM_000546:exon6:c.646G>A:p.V216M | 14.3  2.1 |
| **20** | 46,XY[14] | *ASXL1* NM_015338:exon12:c.1927dupG:p.G642fs  *BRAF* NM_004333:exon15:c.1781A>G:p.D594G  *ETNK1* NM_018638:exon3:c.731A>G:p.N244S  *EZH2* NM_004456:exon18:c.2110+1G>A  *EZH2* NM_004456:exon18:c.2058dupT:p.N687_K688delins* | 7.1  8.7  10.7  8.5  9.8 |
| CE |  | *JAK2* NM_004972:exon14:c.1849G>T:p.V617F  *NRAS* NM_002524:exon2:c.35G>A:p.G12D  *KRAS* NM_033360:exon2:c.34G>C:p.G12R | 2.8  3.1  2.2 |
| **21** | 46,XY[20] | *BCOR* NM_017745:exon4:c.2340dupC:p.T781fs  *CEBPA* NM_001287424:exon1:c.337delC:p.L113fs  *EZH2* NM_004456:exon17:c.2007C>G:p.S669R  *TET2* NM_001127208:exon3:c.2974_2977del:p.C992fs  *TET2* NM_001127208:exon7:c.3909C>G:p.S1303R  *ZRSR2* NM_005089:exon10:c.937G>C:p.G313R | 51.7  48.1  81.5  39.3  42.2  70.5 |
| **23** | 46,XY[21] | *FLT3* NM_004119:exon14:c.1793_1794insCTACGTTG ATTTCAGAGAATATGA:p.E598delinsDYVDFREYE | 6.6 |
| **24** | 46,XY[20] | *ETV6* NM_001987:exon2:c.163+1G>C  *GATA2* NM_032638:exon4:c.953C>T:p.A318V  *ETV6* NM_001987:exon2:c.162_163insCTT:p.L54delinsLL | 11.0  16.8  10.1 |
| **27** | 46,XX,t(9;11)(p21;q23)[15]/46,XX[5] | *NRAS* NM_002524:exon2:c.35G>A:p.G12D | 22.0 |
| **28** | 46,XX[1] | *IDH2* NM_002168:exon4:c.515G>A:p.R172K | 46.1 |
| **32** | 47,XX,+8,der(18)t(3;18)(q26;p11.2)[1]/46,XX[29] | *NPM1* NM_002520:exon11:c.859_860insTCTG:p.L287fs (TypA) | 69.27 (qPCR) |
| **33** | 46,XY[20] | *ASXL1* NM_015338:exon12:c.1888_1910del:p.H630fs  *BCOR* NM_017745:exon4:c.880dupA:p.S294fs  *RUNX1* NM_001754:exon9:c.1003_1015del:p.Q335fs  *SRSF2* NM_003016:exon1:c.284C>A:p.P95H  *STAG2* NM_006603:exon22:c.2229G>A:p.W743* | 7.5  13.2  7.9  14.5  17.9 |
| **34** | not available | Negative |  |
| **35** | 46,XX[18] | *JAK2* NM_004972:exon14:c.1849G>T:p.V617F  *SF3B1* NM_012433:exon14:c.1996A>G:p.K666E  *TP53* NM_001126114:exon5:c.476C>T:p.A159V | 46.2  49.3  94.6 |
| **36** | 46,XY[24] | *IDH2* NM_002168:exon4:c.419G>A:p.R140Q  *NPM1* NM_002520:exon11:c.859_860insTCTG:p.L287fs (TypA)  *SRSF2* NM_003016:exon1:c.284C>A:p.P95H | 31.0  7.9  43.1 |
| **38** | 48,XY,+8,+8 [21] | *BCOR* NM_017745:exon15:c.4879C>T:p.R1627*  *BCOR* NM_017745:exon4:c.2382delG:p.G794fs  *FLT3* NM_004119:exon20:c.2503G>A:p.D835N  *IDH2* NM_002168:exon4:c.515G>A:p.R172K  *KIT* NM_000222:exon10:c.1588G>A:p.V530I  *KRAS* NM_033360:exon3:c.179G>A:p.G60D  *PTPN11* NM_002834:exon13:c.1508G>A:p.G503E | 25.0  31.6  2.2  37.6  31.1  10.9  17.7 |
| **39** | 47,XX,+8[6]/46,XX[15] | *BCORL1* NM_021946:exon6:c.3874C>T:p.R1292*  *EZH2* NM_004456:exon17:c.2023A>T:p.N675Y  *EZH2* NM_004456:exon17:c.1969G>T:p.D657Y  *FLT3* NM_004119:exon20:c.2504A>T:p.D835V  *IDH2* NM_002168:exon4:c.419G>A:p.R140Q  *RUNX1* NM_001754:exon9:c.1222_1223insTACG:p.G408fs  *STAG2* NM_006603:exon18:c.1810C>T:p.R604* | 22.6  49.8  43.1  2.3  36.1  17.0  2.3 |
| **40** | not available | *BCOR* NM_001123385:exon12:c.4741G>A:p.D1581N  *DNMT3A* NM_022552:exon9:c.1031T>A:p.L344Q  *NRAS* NM_002524:exon3:c.173C>T:p.T58I  *PTPN11* NM_002834:exon3:c.181G>C:p.D61H  *U2AF1* NM_006758:exon2:c.101C>T:p.S34F | 31.5  31.2  23.6  9.1  30.6 |
| **41** | 44,XY,del(3)(p21),der(5)t(3;5)(p21;p14)del(5)(q14q34),del(6)(q24q26),17,der(21;22)t(21;22)(p11;p11)dup(21)(q11.2q2?2)[7]/44,  idem,i(8)(q10)[10]/45,idem,+22[5]/44,idem,t(11;12)(q2?3;q13)[2]/44,idem,der(4)t(4;15)(q13;q14),del(8)(?p21),-15,+22[1] | *TP53* NM_000546:exon8:c.818G>A:p.R273H | 92.8 |
| **42** | 46,XX[24] | *SRSF2* NM_003016:exon1:c.284C>T:p.P95L  *STAG2* NM_006603:exon27:c.2898_2902del:pT966fs  *TET2* NM_001127208:exon7:c.3893G>A:p.C1298Y | 36.3  21.4  13.6 |
| CE |  | *NRAS* NM_002524:exon2:c.35G>A:p.G12D | 6.1 |
| **43** | 46,XX[15] | *TET2* NM_017628:exon3:c.2926C>T:p.Q976*  *SRSF2* NM_003016:exon1:c.284C>A:p.P95H  *RUNX1* NM_001754:exon6:c.611G>T:p.R204L | 83.5  48.1  45.6 |
| **44** | not available | *ASXL1* NM_015338:exon12:c.2472_2476del:p.E824fs  *CBL* NM_005188:exon9:c.1259G>A:p.R420Q  *CUX1* NM_181552:exon22:c.3535C>T:p.R1179*  *EZH2* NM_004456:exon5:c.394C>A:p.P132T  *TET2* NM_001127208:exon6:c.3629T>C:p.L1210P  *TET2* NM_001127208:exon6:c.3782G>A:p.R1261H | 16.2  10.8  16.2  8.0  20.5  22.6 |
| **45** | not available | *RUNX1* NM_001754:exon8:c.952dupT:p.S318fs  *SRSF2* NM_003016:exon1:c.284C>G:p.P95R  *EZH2* NM_004456:exon15:c.1769_1772del:p.C590fs | 35.0  39.0  30.6 |
| **46** | 46,XY[20] | *ASXL1* NM_015338:exon12:c.1888_1910del:p.H630fs  *BCOR* NM_017745:exon9:c.4038_4039del:p.T1346fs  *RUNX1* NM_001754:exon8:c.941_942del:p.S314fs  *SRSF2* NM_003016:exon1:c.284C>A:p.P95H  *TET2* NM_001127208:exon6:c.3748G>T:p.E1250* | 26.2  80.8  41.1  40.0  46.6 |
| **48** | 47,XY,+8[5]/46,XY[6]/46,XX[1] | Negative |  |
| **49** | 46,XY[2] | *TP53* NM_000546:exon7:c.746_747delinsA:p.R249fs  *RUNX1* NM_001754:exon8:c.952_953insTG:p.S318fs  *FLT3*-ID^high^ | 4.7  9.6  ratio: 0.89 |
| CE | 48,X,der(Y)t(Y;13)(q12;q2?2),t(1;8)(p34;p23),der(2)t(2;4)(q3?4;?p12),dup(3)(q26q28),-13,der(17)t(11;17)(q2?1;p13),+20,+21,+mar[25] |  |  |
| **50** | 46,XY[20] | *IDH2* NM_002168:exon4:c.419G>A:p.R140Q  *SRSF2* NM_003016:exon1:c.284C>A:p.P95H  *KMT2A*-PTD | 2.9  3.2  n.a |
| **51** | 46,XY[10] | *BCOR* NM_017745:exon4:c.2626_2633del:p.F876fs  *BCORL1* NM_021946:exon10:c.4620_4621insAGAAG:p.D1540fs  *BCORL1* NM_021946:exon10:c.4621C>A:p.H1541N  *RUNX1* NM_001754:exon4:c.319C>T:p.R107C | 9.8  8.7  8.4  5.7 |
| **52** | 46,XX[22] | Negative |  |
| **53** | 46,XX[20] | *DNMT3A* NM_022552:exon23:c.2645G>A:p.R882H  *NPM1* NM_002520:exon11:c.859_860insTCTG:p.L287fs (TypA)  *SF3B1* NM_012433:exon15:c.2098A>G:p.K700E  *FLT3*-ITD^low^ | 48.9  43.7  46.1  ratio: 0.17 |
| CE | 47,XX,+15[3]/46,XX[19]  Trisomy 11/11q in FISH | *PRPF8* NM_006445:exon29:c.4631G>A:p.R1544Q | 3.8 |
| **55** | 46,XX[20] | *DNMT3A* NM_022552:exon19:c.2194T>C:p.F732L  *KRAS* NM_033360:exon2:c.34G>T:p.G12C  *NPM1* NM_002520:exon11:c.859_860insTCTG:p.L287fs (TypA)  *PTPN11* NM_002834:exon3:c.205G>A:p.E69K  *PTPN11* NM_002834:exon3:c.215C>T:p.A72V | 22.4  3.8  30.9  18.9  6.4 |
| **56** | 46,XX,t(2;3)(q2?3;p1?3),del(12)(p12)[15]/46,XX[4]//46,XY[4] | *ASXL1* NM_015338:exon12:c.1927dupG:p.G642fs  *EZH2* NM_004456:exon5:c.446T>A:p.L149Q  *RUNX1* NM_001122607:exon3:c.521G>A:p.R174Q  *SETBP1* NM_015559:exon4:c.2602G>A:p.D868N | 23.2  27.5  1.6  1.0 |
| CE |  | *FLT3-ITD*  *NRAS* NM_002524:exon2:c.37G>C:p.G13R | High  3.6 |
| **57** | 46,XX[15] | *ASXL1* NM_015338:exon12:c.4122dupG:p.V1374fs  *BCOR* NM_001123385:exon4:c.2514dupC:p.K839fs  *IDH2* NM_002168:exon4:c.419G>A:p.R140Q  *SRSF2* NM_003016:exon1:c.284C>T:p.P95L  *STAG2* NM_006603:exon26:c.2775+2T>G  MLL-PTD (e9e3), MLL-PTD (e10e3), MLL-PTD (e11e3) | 35.4  6.1  25.6  47.6  64.5  n.a |
| CE |  | *BCOR* NM_001123385:exon4:c.2514dupC:p.K839fs  *NRAS* NM_002524:exon2:c.37G>C:p.G13R  *KMT2A-PTD* | 6.1  18.9  N.A |
| **58** | 46,XY,del(5)(q11),der(6)t(6;13)(p?21;q14),del(13)(q?22q?34),del(13)(q14),der(17)del(17)(p11)del(17)(q11q22)[17]/  46,XY,+8,der(8;9)(q10;q10)x2,+9[2]/46,XY[2] | *JAK2* NM_004972:exon14:c.1849G>T:p.V617F  *TP53* NM_000546:exon5:c.380C>T:p.S127F | 90.3  81.0 |
| **59** | 47,XY,+8[5]/47,XY,del(9)(q21q22),+11[13]/48,idem,+13[6] | *U2AF1* NM_006758:exon2:c.101C>T:p.S34F  *IDH1* NM_005896:exon4:c.394C>T:p.R132C  *BCOR* NM_001123385:exon12:c.4741+1G>A  *FLT3* NM_004119:exon20:c.2503G>T:p.D835Y | 26.0  1.0  34.0  4.0 |
| **60** | not available | *TP53* NM_000546:exon6:c.578A>T:p.H193L  *DNMT3A* NM_022552:exon23:c.2729C>T:p.A910V  *NRAS* NM_002524:exon3:c.181C>A:p.Q61K | 67.0  5.0  30.0 |
| **63** | 45,X,der(X)t(X;14)(q2?3;q32),der(4)t(4;12)(p16;q15),der(5)t(5;7)(q12;?p15),der(7;12)(p10;q10)del(7)(p?15)del(12)(q15),+8,  der(11;14)t(11;14)(p15;p11)?dup(11)(q14q24)t(11;X)(q25;q2?3),der(20)(20pter->20q13::5q12->5q12::5q?23->5q12::5q35 >5qter)[23] | Negative |  |
| CE | *RUNX1*-, 16q- in FISH, MLL amplification |  |  |
| **64** | 46,XX,t(14;18)(q31;q12)?c[21] | *FLT3* NM_004119:exon20:c.2503G>T:p.D835Y *KRAS* NM_033360:exon2:c.71T>A:p.I24N  *NPM1* NM_002520:exon11:c.863_864insTCGA:p.W288f | 15.1  12.2  22.8 |
| **65** | 44,XY,del(1)(p12p36),der(5)t(5;13)(q1?5;q14),-7,-12,der(13)t(1;13)(p1?3;q14),der(16)t(12;16)(q12;q24)[16]/45,XY,-5,-12,+mar[4] | *TET2* NM_001127208:exon11:c.5500C>T:p.Q1834*  *TET2* NM_001127208:exon6:c.3595-2A>G  *TP53* NM_000546:exon5:c.559+1G>A  *TP53* c.265_266insTGCACCAGCCC:p.P89fs | 1.2  12.9  11.9  7.9 |
| CE | 43,XY,del(1)(p12p36),-4,der(5)t(5;13)(q1?5;q14),-7,der(12)t(12;18)(p12;q11.2),der(13)t(1;13) (p1?3;q14),-16,-18,der(19)t(4;19)(q?24;p12),der(19)t(5;19)(q31;p12),+19[2]/46,XY[4] |  |  |
| **66** | 47,XY,der(21)t(9;21)(p2?2;q22),+der(21)t(9;21)(p2?2;q22)[14]/46,XY,t(11;19)(p15;p13)[4]/46,XY[4] | *IDH1* NM_005896:exon4:c.211G>A:p.V71I  *JAK2* NM_004972:exon14:c.1849G>T:p.V617F  *RUNX1* NM_001122607:exon3:c.512A>G:p.D171G  *RUNX1* NM_001122607:exon2:c.320C>T:p.A107V  *SRSF2* NM_003016:exon1:c.284C>A:p.P95H  *TET2* NM_001127208:exon3:c.1334delT:p.L445fs  *U2AF1* NM_006758:exon6:c.470A>G:p.Q157R | 28.8  93.0  52.2  17.8  46.3  40.4  21.8 |
| **67** | 46,XX[20] | *GATA2* NM_032638:exon6:c.1168_1170del:p.390_390del  *U2AF1* NM_006758:exon6:c.470A>G:p.Q157R | 11.6  13.4 |
| **68** | 46,XY[10] | *BCOR* NM_001123385:exon13:c.4816dupT:p.C1606fs  *RUNX1* NM_001122607:exon1:c.86dupT:p.L29fs  *STAG2* NM_006603:exon22:c.2263A>T:p.K755*  *TET2* NM_001127208:exon11:c.4657_4660del:p.Q1553fs  *ZRSR2* NM_005089:exon7:c.535G>A:p.G179R  *BCOR* NM_001123385:exon6:c.3199C>A:p.L1067M | 9.1  6.9  6.1  4.2  8.3  1 |
| **69** | 46,XY[22] | *BCOR* NM_017745:exon7:c.3270_3274delinsT:p.(F1092fs)  *BCORL1* NM_021946:exon3:c.1916G>T:p.R639L  *RUNX1* NM_001754:exon9:c.1011delC:p.P337fs  *SETBP1* NM_015559:exon4:c.3436C>T:p.R1146W  *SETBP1* NM_015559:exon4:c.3473A>G:p.H1158R | 33.5  29.4  19.5  6.6  16.1 |
| **70** | 44,XY,dic(5;6)(q12;p25),der(7)t(7;20)(q2?2;?p12),-13,r(20)(p12q?11.2)[8]/46,XY[12] | *DNMT3A* NM_153759:exon5:c.464T>C:p.L155P  *PPM1D* NM_003620:exon6:c.1451T>G:p.L484*  *TP53* NM_000546:exon10:c.1024C>T:p.R342*  *TP53* NM_001126114:exon7:c.761T>G:p.I254S | 13.8  11.4  25.6  20.9 |
| **71** | 45,XX,-7[19]/46,idem,+21[5] | *DNMT3A* NM_022552:exon23:c.2645G>A:p.R882H | 35.9 |

Supplementary Table 5. Immunophenotypic characterization at initiation by flow cytometry of leukemic blasts from the 22 MDS and AML patients included in BH3 profiling analysis treated with aza/ven.

| ID | Immunophenotype |
| --- | --- |
| 3 | CD45(+), CD34+, CD13+, CD33+, CD38+, CD117+, HLA-DR- |
| 6 | CD45(+), CD34+, CD13+, CD33+, CD117+, HLA-DR+, CD15- |
| 10 | CD45(+), CD34+, CD13+, CD33+, CD117+, HLA-DR+ |
| 16 | CD45(+), CD34+, CD13+, CD33-, CD117+, HLA-DR+ |
| 17 | CD45(+), CD34+, CD13+, CD33+, CD117+, HLA-DR+, CD15- |
| 19 | CD45(+), CD34+, CD38+, CD33-, CD117-, CD13(+) |
| 24 | CD45+, CD34+, CD13+, CD33+, CD117+, HLA-DR+, CD15- |
| 27 | CD45(+), CD34+, CD13+, CD33+, CD117(+), HLA-DR(+), CD15+, CD65+, CD4-, CD7- |
| 28 | CD45(+), CD34(+) |
| 33 | CD45(+), CD34+, CD13+, CD33+, CD117+, HLA-DR+, CD34+, CD15-, CD19-, CD4+, MPO+ |
| 41 | CD45+, CD34+, CD117+, CD13+, CD33+, HLA-DR+ |
| 43 | CD45(+), CD34+, CD13+, CD38+, CD33+, CD117+ |
| 44 | CD45(+), CD34+, CD117+, CD15-, CD33+, CD38+, CD117+, HLADR+, CD123+ |
| 49 | CD45(+), CD34+, CD13+, CD33+, CD117+, HLA-DR+, CD38+, cyMPO+, CD4-, cyCD3-, cyCD79a- |
| 50 | CD45(+), CD34+/-, CD13-/+, CD33+, CD117+, HLA-DR+/-, CD15+/-, Flt3/CD135+ |
| 53 | CD45(+), CD34+, CD13+, CD33+, CD117+, HLA-DR+, MPO+, CD4-, CD7- |
| 55 | CD45(+), CD34+/-, CD38+, CD33+, CD117+, CD13-, HLA-DR+ |
| 56 | CD45(+), CD34+, CD13+, CD38+, CD33+, CD15-, HLADR+, CD117+, CD13+, CD33+, CD117+, HLA-DR+ |
| 59 | CD45(+), CD34+, CD13-, CD33+, CD117+, HLA-DR+, CD15+ |
| 60 | CD45(+), CD34+ |
| 63 | CD45(+), CD34+, CD13-, CD33+, CD38+, CD117+, HLA-DR+, CD15-, CD123+, CD10(+), cyMPO(+), CD56(+), CD14(+) |
| 67 | CD45(+), CD34+, CD13(+), CD33+, CD117(+), HLA-DR+, CD15- |

Supplementary Table 6. Demographic, hematological and clinical data from the 22 patients treated with AV therapy.

| **Variable** | **All patients,**  **n=22** | | ***p*-value** |
| --- | --- | --- | --- |
| **Demographics** | | | |
| Gender (M/F), n (ration) | 9/13 | 0.7 | - |
| Median age, years (range) | 73 | 33-83 | **-** |
| **Cytomorphologic subtypes (n=22)** | | | |
| MDS | 6/22 | 27% | **-** |
| AML | 16/22 | 73% | **-** |
| **Treatments** |  |  |  |
| Number aza/ven therapy cycles, median (range) | 3 | 1-17 | **-** |
| **Outcome** | | |  |
| OS from aza/ven initiation, median mo (95% CI) |  |  |  |
| BAD-HRK^low^ (n=10) | 5 | 3-nr | 0.038 |
| BAD-HRK^high^ (n=12) | 14 | 8-nr |  |
| OS from aza/ven initiation, median mo (95% CI) |  |  |  |
| Non responders (n=9) | 8 | 3-nr | 0.217 |
| Responders (n=13) | 14 | 4-nr |  |

M, male; F, female; MDS, myelodysplastic neoplasms; AML, acute myeloid leukemia; CMML (chronic myelomonocitic leukemia) only for the considered cohort of patients receiving azacitidine in combination with venetoclax (n=23pts). Aza/ven, azacitidine in combination with venetoclax; OS, overall survival; AV; BAD-HRK low/high, patients were categorized into high dependent (≥ mean) and low dependent (<mean); nr, not reached.
