# Supplementary material for: Clonal evolution and apoptosis resistance in myelodysplastic neoplasms and acute myeloid leukemia under treatment: insights from integrative longitudinal profiling
Source: Leukemia. 2025 Sep 19;39(12):3026–36. doi: 10.1038/s41375-025-02756-7 (PMC12634422; doi:10.1038/s41375-025-02756-7)
Supplement: Supplementary file 2 — Supplementary Figures [file 41375_2025_2756_MOESM2_ESM.pdf]

S1

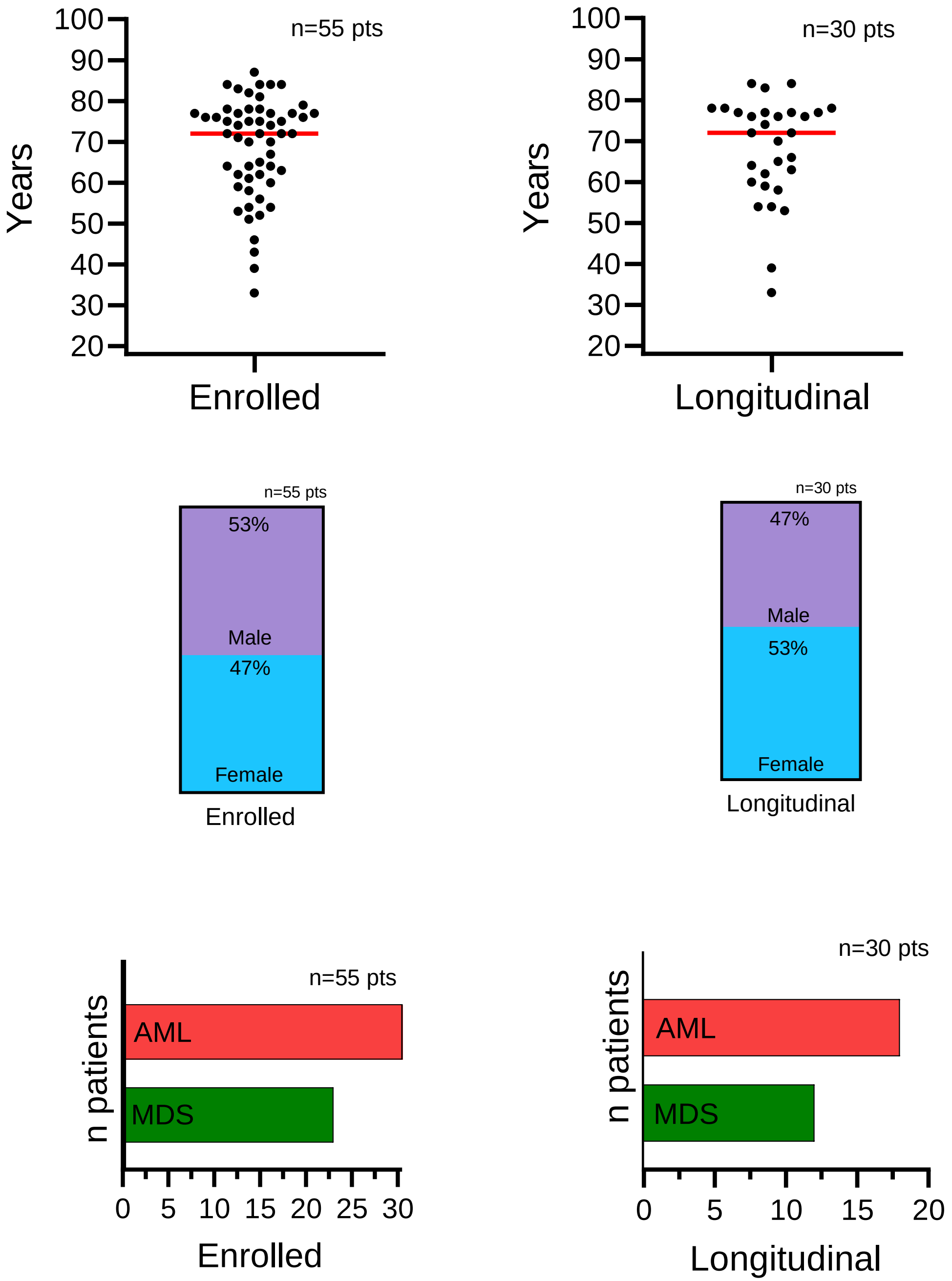

n=55 pts
Years
100
90
80
70
60
50
40
30
20
Enrolled
n=30 pts
Years
Longitudinal
n=55 pts
53%
Male
47%
Female
Enrolled
n=30 pts
47%
Male
53%
Female
Longitudinal
n=55 pts
n patients
AML
MDS
0 5 10 15 20 25 30
Enrolled
n=30 pts
n patients
AML
MDS
0 5 10 15 20
Longitudinal

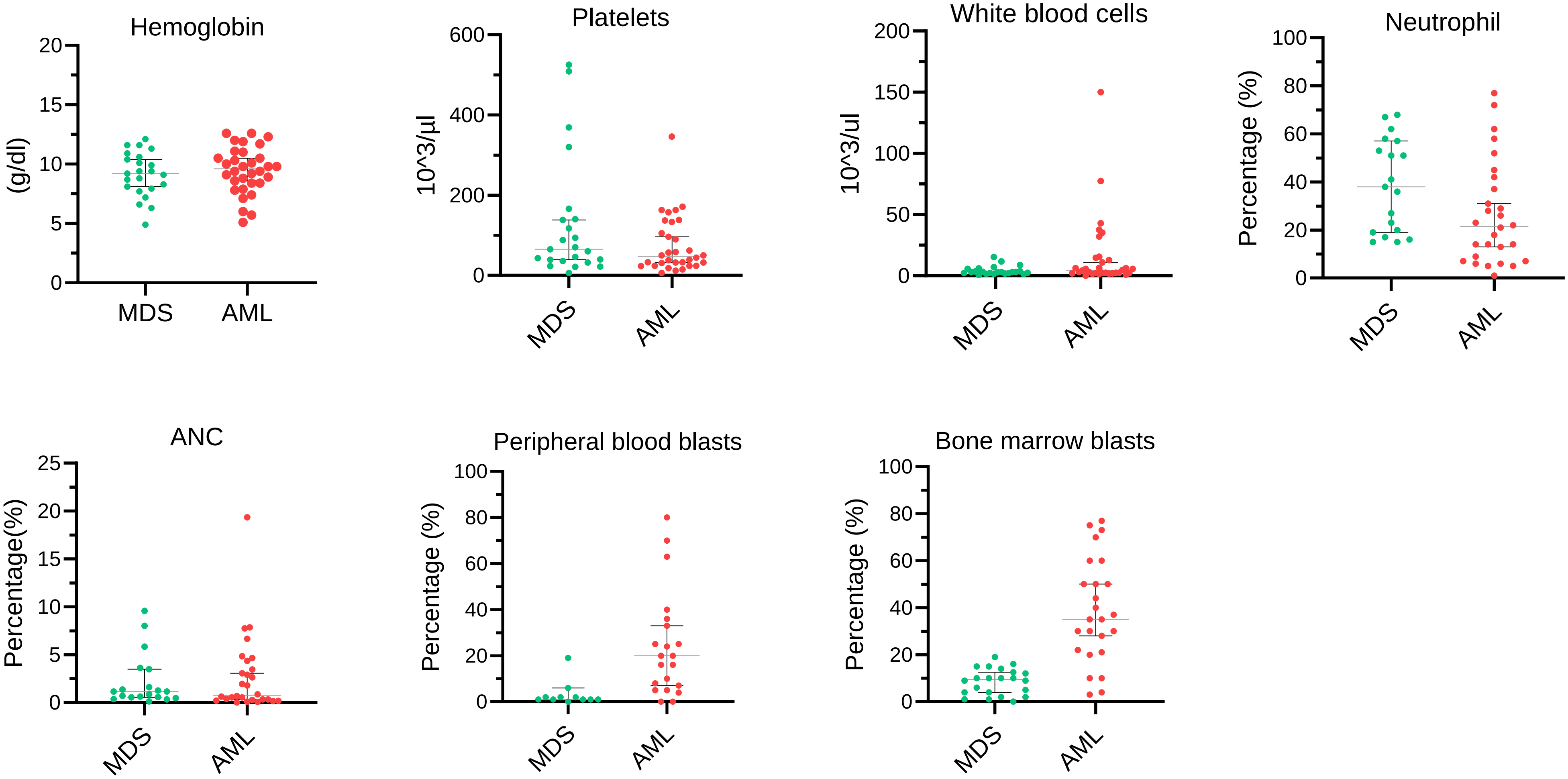
Hemoglobin
(g/dl)
20
15
10
5
0
MDS
AML
Platelets
10^3/µl
600
400
200
0
MDS
AML
White blood cells
10^3/ul
200
150
100
50
0
MDS
AML
Neutrophil
Percentage (%)
100
80
60
40
20
0
MDS
AML
ANC
Percentage(%)
25
20
15
10
5
0
MDS
AML
Peripheral blood blasts
Percentage (%)
100
80
60
40
20
0
MDS
AML
Bone marrow blasts
Percentage (%)
100
80
60
40
20
0
MDS
AML

S3

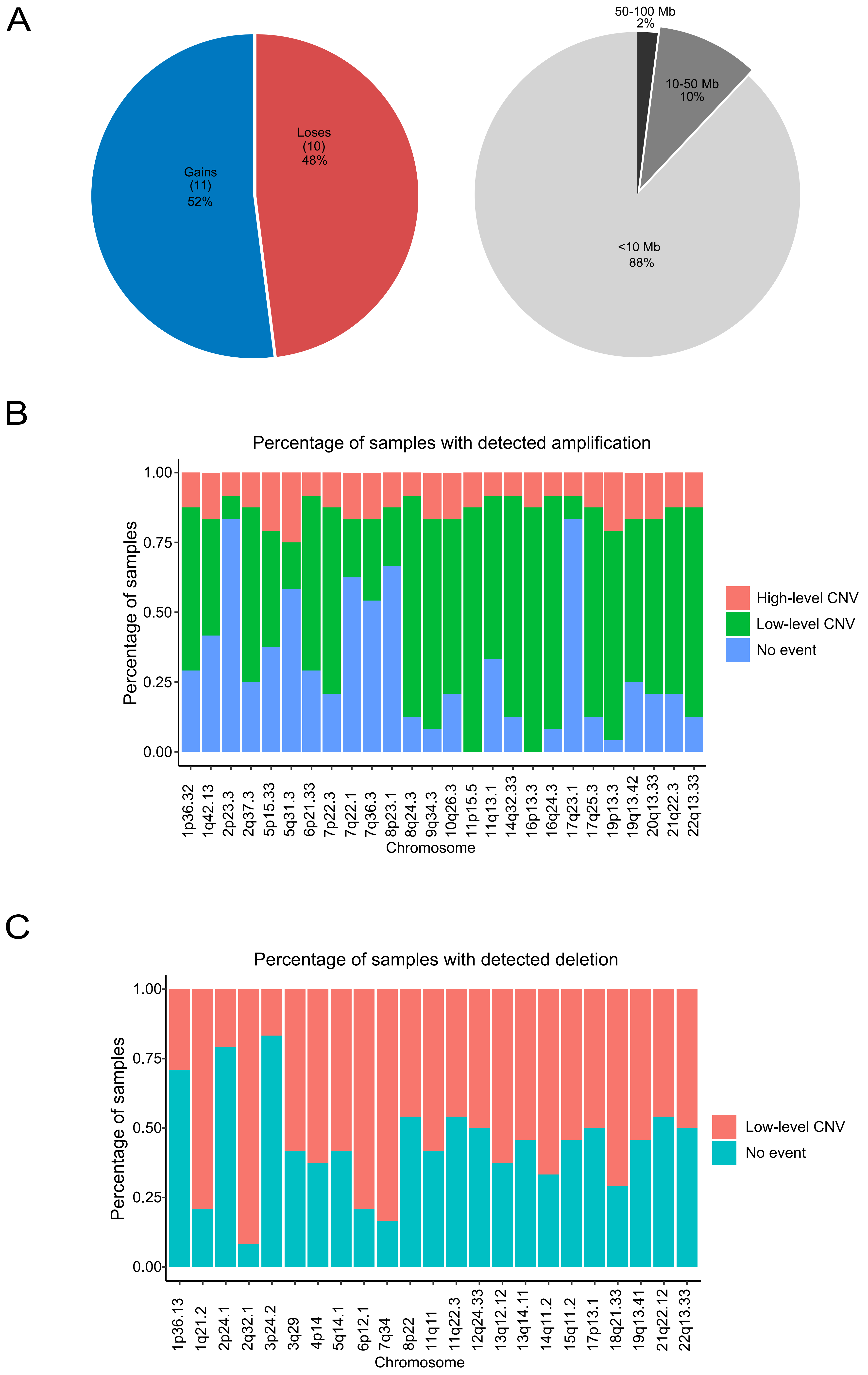

A
Gains
(11)
52%
Loses
(10)
48%
50-100 Mb
2%
10-50 Mb
10%
<10 Mb
88%
B
Percentage of samples with detected amplification
Percentage of samples
1.00
0.75
0.50
0.25
0.00
1p36.32
1q42.13
2p23.3
2q37.3
5p15.33
5q31.3
6p21.33
7p22.3
7q22.1
7q36.3
8p23.1
8q24.3
9q34.3
10q26.3
11p15.5
11q13.1
14q32.33
16p13.3
16q24.3
17q23.1
17q25.3
19p13.3
19q13.42
20q13.33
21q22.3
22q13.33
Chromosome
High-level CNV
Low-level CNV
No event
C
Percentage of samples with detected deletion
Percentage of samples
1.00
0.75
0.50
0.25
0.00
1p36.13
1q21.2
2p24.1
2q32.1
3p24.2
3q29
4p14
5q14.1
6p12.1
7q34
8p22
11q11
11q22.3
12q24.33
13q12.12
13q14.11
14q11.2
15q11.2
17p13.1
18q21.33
19q13.41
21q22.12
22q13.33
Chromosome
Low-level CNV
No event

S4

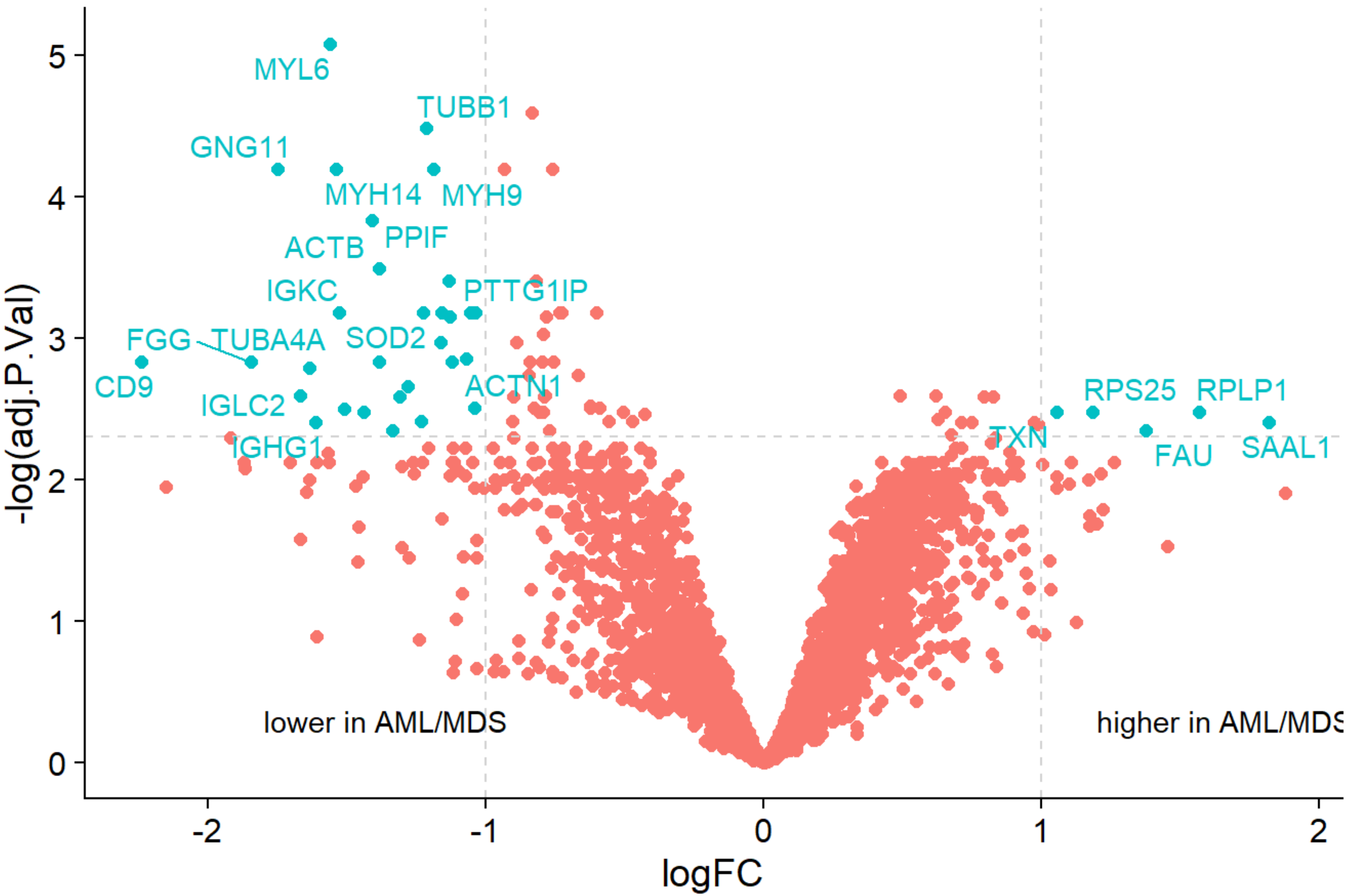
MYL6
TUBB1
GNG11
MYH14
MYH9
PPIF
ACTB
IGKC
PTTG1IP
FGG
TUBA4A
SOD2
CD9
ACTN1
IGLC2
IGHG1
RPS25
RPLP1
TXN
FAU
SAAL1
lower in AML/MDS
higher in AML/MDS
-log(adj.P.Val)
logFC
5
4
3
2
1
0
-2
-1
0
1
2

S5

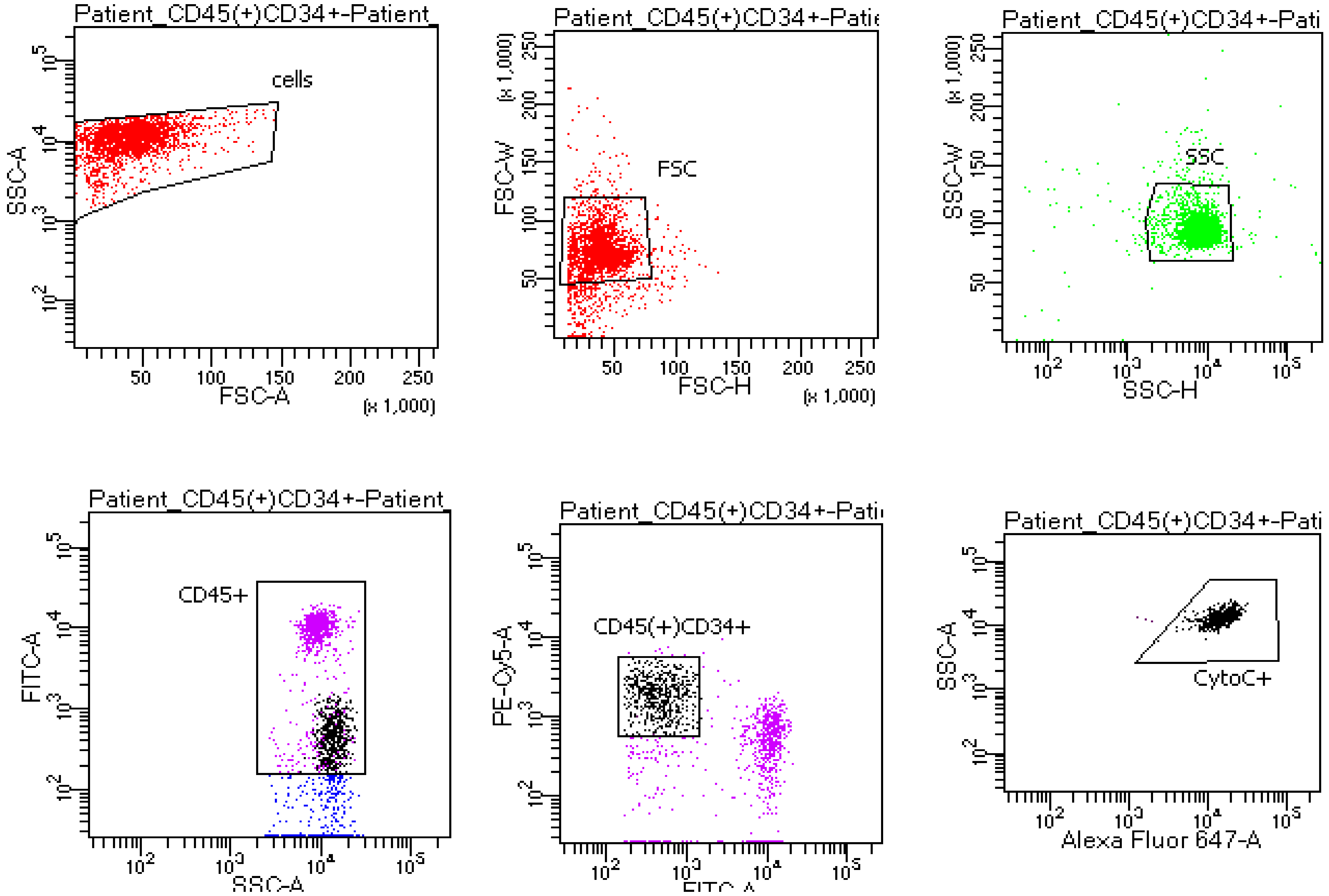
Patient_CD45(+)CD34+-Patient_
cells
SSC-A
FSC-A
(x 1,000)
Patient_CD45(+)CD34+-Patie
FSC
FSC-W
FSC-H
(x 1,000)
Patient_CD45(+)CD34+-Pati
SSC
SSC-W
SSC-H
Patient_CD45(+)CD34+-Patient
CD45+
FITC-A
Patient_CD45(+)CD34+-Pati
CD45(+)CD34+
PE-Cy5-A
Patient_CD45(+)CD34+-Pati
CytoC+
SSC-A
Alexa Fluor 647-A

S6

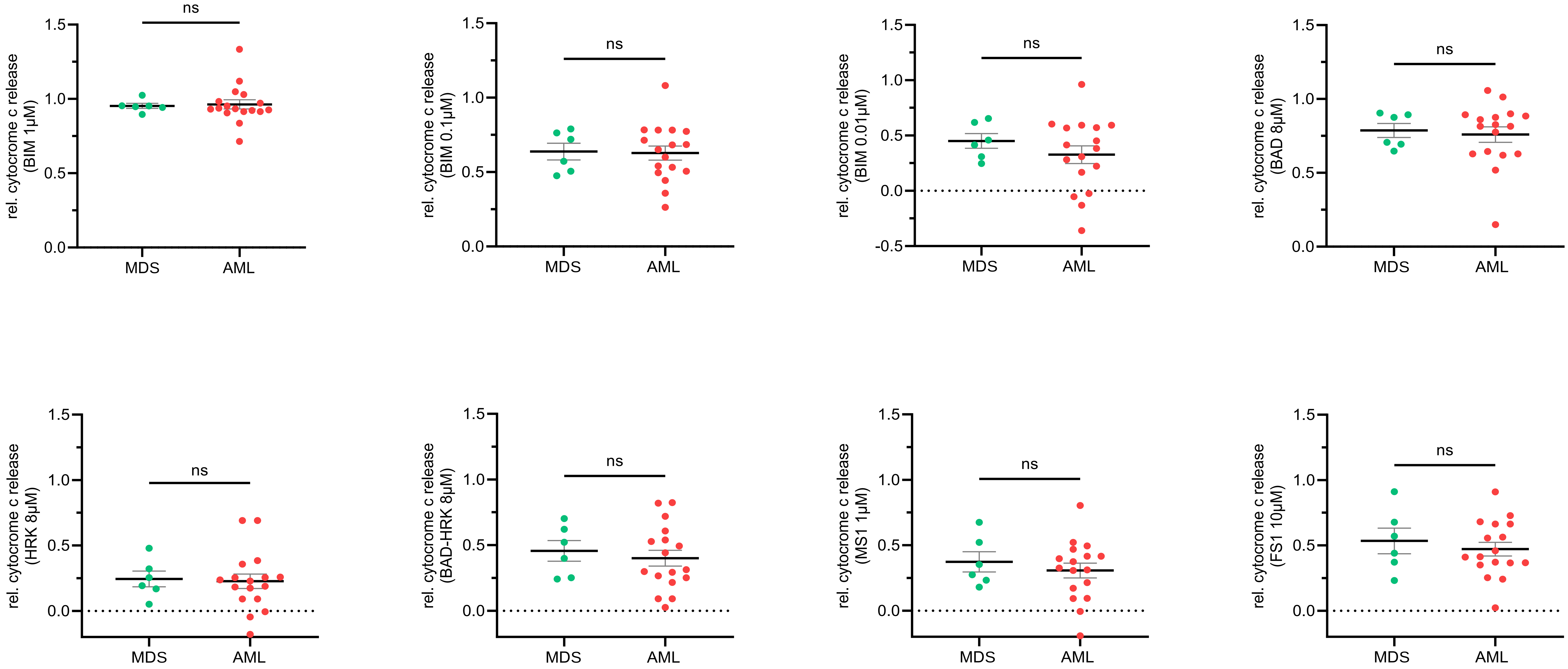
ns
rel. cytocrome c release (BIM 1µM)
rel. cytocrome c release (BIM 0.1µM)
rel. cytocrome c release (BIM 0.01µM)
rel. cytocrome c release (BAD 8µM)
rel. cytocrome c release (HRK 8µM)
rel. cytocrome c release (BAD-HRK 8µM)
rel. cytocrome c release (MS1 1µM)
rel. cytocrome c release (FS1 10µM)
1.5
1.0
0.5
0.0
-0.5
MDS
AML

Supplementary Figure Legend

Figure S1. Main Characteristics of the MDS/AML cohort. a) Age distribution across all the enrolled MDS/AML patients and b) patients we were able to collect longitudinal data. c) Gender distribution across all the enrolled MDS/AML patients and d) patients we were able to collect longitudinal data. e) Distribution of the number of patients included in the study from each type and f) patient we were able to collect longitudinal data. MDS, myelodysplastic neoplasms; AML, acute myeloid leukemia.

Figure S2. Main hematological parameters of the 55 MDS/AML patients. Distribution of peripheral blood counts (hemoglobin, white blood cells, platelets, neutrophil and peripheral blast percentage) and bone marrow blast percentage. MDS, myelodysplastic neoplasms; AML, acute myeloid leukemia.

Figure S3. Types of copy number variants detected in MDS/AML cohort. a) Pie chart on the left shows the distribution of detected gains and losses and on the right, the distribution of CNVs sizes. b-c) Percentage of samples with detected amplification and deletion. The CNV level was determined based on the t-value, which represents the copy number change. The cutoffs classify deviations into no, low, or high levels of amplification or deletion based on the value of t. Amplification: no amplification with t<0.1; low-level with t between 0.1-0.9 and high-level with t>0.9. Deletion: no amplification with t>-0.1; low-level with t between -1.3 and -0.1 and high-level with t< -1.3.MDS, myelodysplastic neoplasms; AML, acute myeloid leukaemia; CNV, copy number variation.

Figure S4. Volcano plot of DEPs. The volcano plot depicts the differential expression of the proteins (lower vs higher) in the MDS/AML cohort vs healthy control samples. MDS, myelodysplastic neoplasms; AML, acute myeloid leukemia.

Figure S5. Flow cytometry gating strategy. Shows CD45(+)CD34+ gating in DMSO settings. CytoC+ gate depicts the cytochrome c retained in the mitochondria.

Figure S6. BH3 peptides distribution across MDS and AML diseases. Each plot shows the difference between MDS and AML patients for each different BH3 profiling peptide. MDS, myelodysplastic neoplasms; AML, acute myeloid leukemia.
